# Small-angle neutron scattering studies on the AMPA receptor GluA2 in the resting, AMPA-bound and GYKI-53655-bound states

**DOI:** 10.1107/S2052252518012186

**Published:** 2018-10-11

**Authors:** Andreas Haahr Larsen, Jerzy Dorosz, Thor Seneca Thorsen, Nicolai Tidemand Johansen, Tamim Darwish, Søren Roi Midtgaard, Lise Arleth, Jette Sandholm Kastrup

**Affiliations:** a Structural Biophysics, X-ray and Neutron Science, The Niels Bohr Institute, University of Copenhagen, Denmark; b Biostructural Research, Department of Drug Design and Pharmacology, Faculty of Health and Medical Sciences, University of Copenhagen, Denmark; c National Deuteration Facility, Australian Nuclear Science and Technology Organization, Australia

**Keywords:** ionotropic glutamate receptor, small-angle neutron scattering, agonists, negative allosteric modulators, resting state, Alzheimer’s disease, Parkinson’s disease, epilepsy

## Abstract

The AMPA receptor GluA2 belongs to the family of ionotropic glutamate receptors, which are responsible for most of the fast excitatory neuronal signalling in the central nervous system. These receptors are important for memory and learning, but have also been associated with brain diseases such as Alzheimer’s disease and epilepsy. Today, one drug is on the market for the treatment of epilepsy targeting AMPA receptors, *i.e.* a negative allosteric modulator of these receptors. Recently, crystal structures and cryo-electron microscopy (cryo-EM) structures of full-length GluA2 in the resting (apo), activated and desensitized states have been reported. Here, solution structures of full-length GluA2 are reported using small-angle neutron scattering (SANS) with a novel, fully matched-out detergent. The GluA2 solution structure was investigated in the resting state as well as in the presence of AMPA and of the negative allosteric modulator GYKI-53655. In solution and at neutral pH, the SANS data clearly indicate that GluA2 is in a compact form in the resting state. The solution structure resembles the crystal structure of GluA2 in the resting state, with an estimated maximum distance (*D*_max_) of 179 ± 11 Å and a radius of gyration (*R*_g_) of 61.9 ± 0.4 Å. An *ab initio* model of GluA2 in solution generated using *DAMMIF* clearly showed the individual domains, *i.e.* the extracellular N-terminal domains and ligand-binding domains as well as the transmembrane domain. Solution structures revealed that GluA2 remained in a compact form in the presence of AMPA or GYKI-53655. At acidic pH only, GluA2 in the presence of AMPA adopted a more open conformation of the extracellular part (estimated *D*_max_ of 189 ± 5 Å and *R*_g_ of 65.2 ± 0.5 Å), resembling the most open, desensitized class 3 cryo-EM structure of GluA2 in the presence of quisqualate. In conclusion, this methodological study may serve as an example for future SANS studies on membrane proteins.

## Abstract 2

In this study, the behaviour of the detergent-solubilized tetrameric, full-length ionotropic glutamate receptor GluA2 in solution was investigated using small-angle neutron scattering. It was found that the GluA2 solution structure is preferentially in a compact form in the resting state as well as in the presence of AMPA and of the negative allosteric modulator GYKI-53655.

## 1. Introduction   

Glutamate is the major excitatory neurotransmitter in the central nervous system (CNS) and mediates its function through interaction with metabotropic G protein-coupled receptors (mGluRs) and ionotropic glutamate receptors (iGluRs). Located in the cell membrane at the synapse, the iGluRs mediate fast synaptic transmission in the CNS and have an important role in memory and learning (Sachser *et al.*, 2017 ▸). However, these receptors have also been associated with brain diseases or disorders, for example epilepsy, Parkinson’s disease, Alzheimer’s disease, depression and stroke (Lee *et al.*, 2016 ▸). Therefore, the iGluRs are considered to be important targets for intervention by medicines: for example, the drugs memantine used for the treatment of Alzheimer’s disease and perampanel used for the treatment of epilepsy both target the iGluRs.

**Figure 1:**
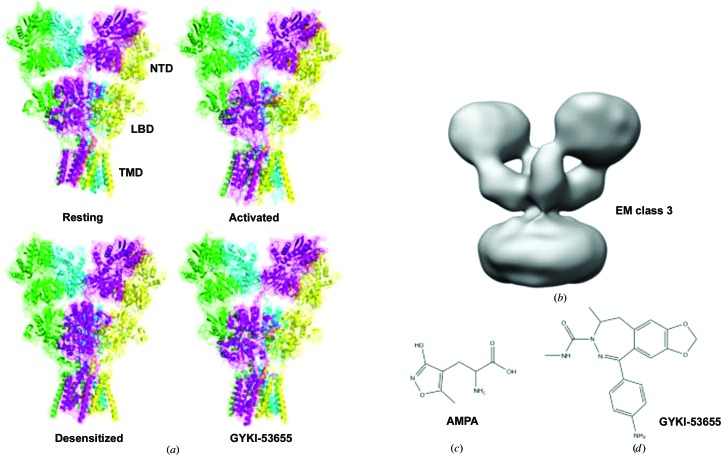
Structures. (*a*) GluA2 in the resting (apo) state (PDB entry 4u2p; Dürr *et al.*, 2014 ▸), the activated state (PDB entry 5weo; Twomey *et al.*, 2017 ▸ *b*), the desensitized state (PDB entry 5vhz; Twomey *et al.*, 2017*a* ▸) and with a negative allosteric modulator bound (PDB entry 5l1h; Yelshanskaya *et al.*, 2016 ▸). The N-terminal domain (NTD), ligand-binding domain (LBD) and transmembrane domain (TMD) are indicated in the figure. Parts of the TMD have not been modelled in the structures. (*b*) The EM class 3 structure (EMDataBank EMD-2688; Meyerson *et al.*, 2014 ▸). (*c*) The agonist AMPA. (*d*) The negative allosteric modulator GYKI-53655.

The members of the iGluR family have been divided into four classes: the α-amino-3-hydroxy-5-methylisoxazole-4-propionate (AMPA) receptors, the kainate receptors, the *N*-methyl-d-aspartate (NMDA) receptors and the delta receptors (Traynelis *et al.*, 2010 ▸). iGluRs form tetrameric ion channels composed of either identical subunits (homomeric receptors) or different subunits (heteromeric receptors). The AMPA receptors consist of subunits GluA1–GluA4, of which the GluA2 subunit has been the most studied. The GluA2 subunit is composed of four domains (Fig. 1 ▸ *a*): the extracellular N-terminal domain (NTD; also abbreviated ATD) followed by the ligand-binding domain (LBD), the transmembrane domain (TMD) forming the ion channel and the cytosolic C-terminal domain (CTD; not included in structures).

The iGluRs have been shown to adopt various conformational states upon activation and inactivation (Fig. 1 ▸ *a*). The first X-ray structure of a full-length homomeric GluA2 was published in 2009 (Sobolevsky *et al.*, 2009 ▸). This structure was of GluA2 with a competitive antagonist bound. Structures of GluA2 in different states followed, for example with agonists and positive allosteric modulators (see, for example, Dürr *et al.*, 2014 ▸) and with negative allosteric modulators (for example perampanel and GYKI-53655; Yelshanskaya *et al.*, 2016 ▸), as well as of GluA2 in the resting (apo) state (Dürr *et al.*, 2014 ▸; Yelshanskaya *et al.*, 2016 ▸). Recently, the first structures of GluA2 in the activated state and in the desensitized state (an inactive form of the receptor where glutamate is still bound) have been reported using cryo-electron microscopy (cryo-EM; Twomey *et al.*, 2017*a* ▸,*b* ▸). To date, approximately 40 full-length GluA2 structures have been deposited in the Protein Data Bank (PDB; http://www.rcsb.org), of which half were determined by X-ray crystallography (Sobolevsky *et al.*, 2009 ▸; Chen *et al.*, 2014 ▸; Dürr *et al.*, 2014 ▸; Yelshanskaya *et al.*, 2014 ▸, 2016 ▸) and the other half by electron microscopy (Meyerson *et al.*, 2014 ▸; Twomey *et al.*, 2016 ▸, 2017*a* ▸,*b* ▸; Zhao *et al.*, 2016 ▸; Chen *et al.*, 2017 ▸). As can be seen in Fig. 1 ▸(*a*), the X-ray structures all represent compact and also rather similar structures, whereas structures with more open extracellular domains have been reported using cryo-EM, for example GluA2 in complex with the agonist quisqualate (class 3; Fig. 1 ▸ *b*). The structure of GluA2 with quisqualate was considered to represent a desensitized state of the receptor (Meyerson *et al.*, 2014 ▸).

Here, we report small-angle neutron-scattering (SANS) data on detergent-solubilized full-length GluA2 (GluA2cryst with deletion of the CTD; Sobolevsky *et al.*, 2009 ▸) using a novel matched-out deuterated *n*-dodecyl-β-d-maltopyranoside (DDM; Midtgaard *et al.*, 2018 ▸). The matched-out DDM ensures that only GluA2 contributes to the measured SANS signal. GluA2 was investigated in its resting state, in the presence of the agonist AMPA (Fig. 1 ▸ *c*) and with the negative allosteric modulator GYKI-53655 (Fig. 1 ▸ *d*). We show that GluA2 in solution at neutral pH primarily adopts a tetrameric compact structure that resembles the X-ray and cryo-EM structures, whereas acidic pH leads to a more open structure. To our knowledge, this is the first time that the structure of full-length GluA2 has been studied as a detergent-solubilized protein in solution. It is also the first time that the structural effect of the binding of AMPA to full-length GluA2 has been studied.

## 2. Methods   

### 2.1. Expression and purification of GluA2   

The GluA2cryst construct was kindly provided by E. Gouaux (Sobolevsky *et al.*, 2009 ▸). The receptor was expressed in the HEK293F cell line and purified as described previously (Midtgaard *et al.*, 2018 ▸). In brief, the membranes were isolated by resuspending the cell pellet in a buffer consisting of 20 m*M* Tris pH 8.0, 150 m*M* NaCl, 45 m*M* DDM (Anatrace) and protease inhibitors (Roche). The supernatant was supplemented with 50 m*M* imidazole, mixed with TALON metal-affinity resin (Clontech) and rotated overnight. The receptor was eluted using the same buffer but containing 250 m*M* imidazole and 1 m*M* DDM. GFP and the His tag were removed by adding thrombin (Sigma, catalogue No. T4393) overnight at 4°C. Finally, the receptor was purified by size-exclusion chromatography on a Superose 6 column (GE Healthcare) and the peak fractions were flash-frozen in liquid nitrogen.

### 2.2. Solvent and detergent exchange   

Deuterated DDM was synthesized to match the scattering length density of D_2_O in both the detergent head and tail, as described by Midtgaard *et al.* (2018 ▸). Purified GluA2 in H_2_O-based buffer (20 m*M* Tris pH 8.0, 150 m*M* NaCl, 1 m*M* DDM Anagrade (Anatrace) was applied onto a Superose 6 10/300 GL (GE Healthcare) column equilibrated with D_2_O-based buffer (20 m*M* Tris–DCl pH 7.5, 100 m*M* NaCl) with 0.5 m*M* deuterated DDM to exchange solvent and detergent and to obtain matched-out conditions in which only the protein is visible. The exchange was performed at 5°C at a flow rate of 0.25 ml min^−1^ to ensure full exchange (Midtgaard *et al.*, 2018 ▸).

### 2.3. Addition of ligands   

The agonist AMPA was dissolved in the abovementioned D_2_O-based buffer to give a 100 m*M* stock solution with a pH of 4.2. The stock solution was added to GluA2 in D_2_O-based buffer by gentle mixing in the SANS cuvettes to obtain one sample with 1 m*M* AMPA pH 7.5 and one with 10 m*M* AMPA pH 5.5. GYKI-53655 was dissolved in hydrogenated DMSO to give a 100 m*M* stock solution and was added to GluA2 in the D_2_O-based buffer to obtain a sample of GluA2 with 1 m*M* GYKI-53655. The protein concentrations were measured as 0.20 mg ml^−1^ (0.54 µ*M*) for the apo sample, 0.31 mg ml^−1^ (0.84 µ*M*) for the 1 m*M* AMPA and the 1 m*M* GYKI-53655 samples, and 0.17 mg ml^−1^ (0.46 µ*M*) for the 10 m*M* AMPA sample. The concentrations were determined by UV absorption at 280 nm using a NanoDrop 1000 (Thermo Fisher Scientific) for the 1 m*M* AMPA and the 1 m*M* GYKI-53655 samples, using an extinction coefficient of 519 100 *M* ^−1^ cm^−1^ as calculated from the construct sequence using *ProtParam* from ExPASy. The concentrations of the apo sample and the 10 m*M* AMPA sample were determined using a QuantiPro BCA assay (Sigma).

### 2.4. SANS data collection   

SANS data were collected using the KWS-1 SANS instrument at FRM II, MLZ, Munich with a neutron wavelength λ of 5.0 Å and a wavelength spread Δλ/λ of 10% (FWHM). Three instrumental settings were used, with collimation and sample-to-detector distances of 4 and 1.5 m, of 4 and 4 m and of 8 and 8 m, respectively, to cover a nominal *q*-range of 0.006–0.3 Å^−1^, where *q* = 4πsin(θ)/λ and θ is half of the scattering angle. Samples were measured in 2 mm Hellma quartz cuvettes at 10°C. The data were reduced according to the standard procedures of the beamline (Feoktystov *et al.*, 2015 ▸), *i.e.* azimuthally averaged, absolute-calibrated with Plexiglass as a standard and background-subtracted using the *QtiKWS* software to yield the reduced scattering intensity *I*(*q*) in units of cm^−1^. Owing to parasitic scattering at high *q*, the data sets were truncated for *q* > 0.2 Å^−1^. The overlap between the three settings was optimized by multiplying the data from the 4/1.5 m setting and the 8/8 m setting by factors close to unity. This optimization was performed with an implementation of the indirect Fourier transformation (IFT) method (Glatter, 1977 ▸), which allows the multiplication factors to be varied to obtain the best fit to the data. The program used was obtained from Jan Skov Pedersen, Aarhus University, Denmark.

### 2.5. Model for SANS data analysis   

An initial inspection of the obtained data suggested the presence of small fractions of higher order oligomers of GluA2 in some of the samples, besides the expected GluA2 tetramers. Models were therefore developed that allowed this effect to be included and each data set was evaluated by fitting the following four different models. Model 1 compared the data directly with the theoretical scattering from relevant GluA2 atomic structures from the Protein Data Bank, *i.e.* GluA2 in the resting state (PDB entry 4u2p; X-ray; Dürr *et al.*, 2014 ▸), the activated state (PDB entry 5weo; cryo-EM; Twomey *et al.*, 2017*a* ▸), the desensitized state (PDB entry 5vhz; cryo-EM; Twomey *et al.*, 2017*a* ▸) and the negative allosteric modulator-inhibited state (PDB entry 5l1h; X-ray; Yelshanskaya *et al.*, 2016 ▸) (Fig. 1 ▸ *a*). In addition, the data were compared with the desensitized GluA2 class 3 cryo-EM structure with quisqualate determined at 22.9 Å resolution by Meyerson *et al.* (2014 ▸) and deposited in the EMDataBank (EMD-2688; Fig. 1 ▸ *b*). In order to calculate the scattering from the class 3 EM structure, an approximate atomic model had to be generated. This was performed by manually fitting an atomic structure of GluA2 in the desensitized state (PDB entry 5vhz) into the EM density map using *UCSF Chimera* (Pettersen *et al.*, 2004 ▸) to generate a class 3 EM atomic structure. The model contains no detergents and is thus directly comparable with the obtained SANS data. This approximate atomic structure was then used to calculate the theoretical scattering from the EM class 3 state. Model 2 was a linear combination of single tetrameric GluA2 in one of the aforementioned states and random oligomers of tetrameric GluA2 in the same state. For the description of the oligomers, the GluA2 tetramers were assumed to be randomly oriented with respect to each other and could thus be modelled as mass fractals (as described below). Model 3 was a linear combination of scattering from one of the atomic structures from model 1 and the desensitized GluA2 class 3 EM structure. Model 4 was a linear combination of the scattering from the atomic structures, of fractal oligomers and of the generated class 3 EM atomic structure, *i.e.* a combination of models 2 and 3.

The models were assessed using the *F*-test based on the reduced χ^2^ values, with a significance criteria of *P* < 5%. The reduced χ^2^ is defined as χ_r_^2^ = χ^2^/*f*, where *f* is the number of degrees of freedom, given in terms of the number of data points *N* and the number of model parameters *K* as *f* = *N* − *K*. χ^2^ is defined as
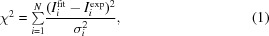
where *I_i_* ^exp^ and σ_*i*_ are the *i*th experimental intensity and error, respectively, and *I_i_* ^fit^ is the *i*th intensity from the model fit. Residual plots are also shown to ease visual comparison of the data and fit, with (Δ*I*/σ)_*i*_ = (*I_i_* ^fit^ − *I_i_* ^exp^)/σ_*i*_.

#### 2.5.1. Model 1   

The scattering intensity was given in terms of the prefactor *C*, the background *B* and the form factor *P*(*q*):
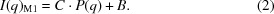
The form factor *P*(*q*) was calculated directly from the relevant atomic structures in PDB format using the software *CaPP* (see §[Sec sec2.5.5]2.5.5 for further details). The prefactor was given as *C* = *K* · *n* · Δ*b* ^2^, where *K* is a correction factor for the protein concentration measurement, *n* is the molar concentration and Δ*b* is the excess scattering length of the protein. *K* and *B* were fitted, *n* was measured by UV absorption and Δ*b* was found by summing atomic scattering lengths (provided, for example, by NIST; https://www.ncnr.nist.gov/resources/n-lengths/) and subtracting the total scattering length of the corresponding excluded water volume.

#### 2.5.2. Model 2   

The random oligomers were described as mass fractals of GluA2 using a previously developed approach (Malik *et al.*, 2012 ▸), which applies the structure factor for fractals of spherical subunits derived by Teixeira (1988 ▸),
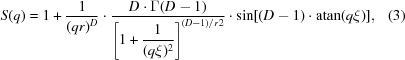
where Γ is the gamma function, *D* is the dimensionality of the fractal (1 < *D* < 3), *r* is the mean distance between the fractal subunits and ξ is the correlation length of the fractal oligomers, which is directly related to the radius of gyration *R*_g_ of the oligomers (Teixeira, 1988 ▸),
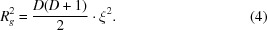



The tetrameric GluA2 subunits were assumed to be randomly oriented with respect to each other in the fractal oligomer. This was taken into account by the decoupling approximation (Kotlarchyk & Chen, 1983 ▸),
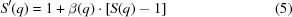
with β(*q*) = 〈ψ(*q*)〉^2^/〈ψ(*q*)^2^〉, where ψ(*q*) is the form-factor amplitude and the angle brackets 〈…〉 denote orientational averaging. As discussed in Høiberg-Nielsen *et al.* (2009 ▸), this can be rewritten as
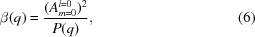
where *A* ^0^_0_ is the amplitude corresponding to the zeroth-order spherical harmonics expansion of ψ(*q*) (Svergun *et al.*, 1995 ▸), which was calculated from the atomic structures. The intensity from one fractal oligomer could then be expressed as a product of *P*(*q*) and *S*′(*q*),
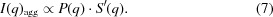



The scattering intensity of model 2 was a linear combination of the scattering from single proteins and fractal oligomers, with γ denoting the fraction of GluA2 molecules in the oligomeric form and *N* denoting the number of GluA2 molecules per oligomer. The intensity could then be written as
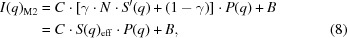
where *S*(*q*)_eff_ is an effective structure factor for the linear combination. *N* is related to *R*_g_, *D* and *r* by the fractal scaling relationship (Sorensen & Roberts, 1997 ▸), 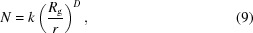
where *k* is the structural coefficient. In this study, we fixed *D* to 2, since the information in the data about the fractals was limited. According to Sorensen & Roberts (1997 ▸), *k* ≃ 1 when *D* ≃ 2, so *k* was furthermore fixed to unity. The mean intermolecular distance *r* of the fractal oligomer was also fixed to equal the radius of a sphere fulfilling *V*_sph_ = *V*_GluA2_, where *V*_GluA2_ was calculated from the protein sequence as a sum of the atomic van der Waals volumes (Svergun *et al.*, 1995 ▸). Hence, the model had a total of four free fitting parameters, *K*, *B*, *R*_g_ and γ, where the last two were directly related to the fractal oligomer. Each data set had 3–5 so-called ‘good parameters’ (Vestergaard & Hansen, 2006 ▸), making it possible to determine all four model parameters well.

#### 2.5.3. Model 3   

It was investigated whether the samples were in an equilibrium between two structural states, namely the atomic structures deposited in the PDB and the class 3 EM structure deposited in the EMDataBank. Denoting the fraction of the intensity coming from the class 3 EM state by α, the intensity could be expressed as
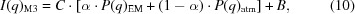
where *P*(*q*)_EM_ and *P*(*q*)_atm_ are the form factors for the class 3 EM structure and one of the atomic structures, respectively. Model 3 had the three parameters *K*, *B* and α.

#### 2.5.4. Model 4   

The fourth model had four contributions to the scattering: GluA2 in one of the atomic structures, GluA2 in the class 3 EM state and fractal oligomers of one of the atomic structures and the class 3 EM structure, respectively. The intensity was given as
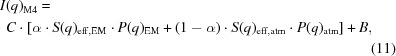
with *S*(*q*)_eff_ as in (8). Model 4 had five free parameters, namely *K*, *B*, *R*_g_, γ and α.

#### 2.5.5. Model implementation   

The models were implemented in *WillItFit* (Pedersen *et al.*, 2013 ▸). Resolution effects were included in the modelling using the resolution function σ_*q*_(*q*) provided by the beamline in the fourth column of the SANS data. The GluA2 form factors were calculated using the C/Python program *CaPP*, which was developed in-house (the source code is freely available at https://github.com/Niels-Bohr-Institute-XNS-StructBiophys/CaPP). *CaPP* is adapted for membrane proteins and allows the inclusion of a hydration layer to only the water-exposed part of the membrane-protein surface and, in the case of SANS studies, exchanges the scattering length of exchangeable H atoms to the average H/D scattering length relevant for the sample. The hydration layer is included as a single layer of water molecules with a density 10% higher than that of bulk water, in accordance with Svergun *et al.* (1998 ▸). The layer is represented by dummy beads, each corresponding to 4.13 water molecules and added to the surface of the protein, except in the region embedded by the core of the DDM micelle. In *CaPP*, the thickness and orientation of the water-depleted layer is either determined using the Orientation of Proteins in Membranes database (https://opm.phar.umich.edu; Lomize *et al.*, 2006 ▸) or defined manually by the user.

### 2.6. Experimental pair distance distribution functions   

The experimental *p*(*r*) functions were calculated by Bayesian indirect Fourier transformation (BIFT) as implemented in *BayesApp* (http://www.bayesapp.org; Hansen, 2012 ▸). Backgrounds were fitted, and for some data sets and fits the regularization parameter and *D*_max_ values were varied manually in the proximity of the automatically determined values, to obtain *p*(*r*) functions with a smooth decay to zero at *D*_max_ and a sensible smoothness. For the data sets for GluA2 in the presence of AMPA at pH 7.5 and of GYKI-53655 slight aggregation was seen. In these cases, a *p*(*r*) function was also calculated for a low-*q* truncated data set in order to limit the effect of aggregation on the refined *p*(*r*) function. The data set for GluA2 in the presence of AMPA at pH 7.5 was truncated after the first four points (*q*_min_ = 0.011 Å^−1^), the data set for GluA2 and AMPA at pH 5.5 was truncated after 14 points (*q*_min_ = 0.019 Å^−1^) and that with GYKI-53655 after five points (*q*_min_ = 0.012 Å^−1^). These were the minimum numbers of data points necessary to remove the ‘tail’ in the *p*(*r*) function for large *r* values. The given experimental values for the radius of gyration *R*_g_, maximal distance in the particle *D*_max_ and forward scattering *I*(0) (Supplementary Table S1; Trewhella *et al.*, 2017 ▸) were estimated from the truncated data sets, since the nontruncated values were influenced by aggregation and thus gave large, and irrelevant, values for *R*_g_ and *D*_max_ that were not comparable with those based on atomic GluA2 structures deposited in the PDB. *R*_g_ and *I*(0) were also determined using Guinier analysis (Supplementary Fig. S1). The data set for the AMPA-bound state of GluA2 at pH 5.5 had no valid Guinier region fulfilling *qR*_g_ < 1.3 (Supplementary Fig. S1*c*) owing to the oligomeric contribution. This led to an overestimation of *R*_g_ and *I*(0) from the Guinier analysis. For the three other samples, the *R*_g_ and *I*(0) values from the Guinier analysis were consistent with those from *p*(*r*) (Supplementary Table S1). In this manuscript, we refer to the *R*_g_ values from *p*(*r*). Likewise, the *R*_g_ and *I*(0) values from *p*(*r*) were used for molecular-weight determination.

### 2.7. Theoretical pair distance distribution functions   

The theoretical *p*(*r*) functions were calculated directly from the atomic structures deposited in the PDB using *CaPP*. Firstly, a hydration shell was added to the structure as described in §[Sec sec2.5]2.5, and the *p*(*r*) functions were then calculated using the positions and scattering lengths of the atoms and water beads. The calculated *p*(*r*) functions had a slowly decreasing asymptotic behaviour for large pair distances *r* because every single atom was included in the calculation. This resulted in a *D*_max_ that was much larger than the experimental value, where the furthermost distances were not detectable. Therefore, in order to obtain a *D*_max_ that could be compared with the experimental value directly, the theoretical *D*_max_ values were calculated with a 1% threshold, *i.e.* the *D*_max_ was defined as the first *r* where *p*(*r*) had decreased to 1% of its maximal value.

### 2.8. *Ab initio* modelling   

Since the scattering from DDM was eliminated by deuteration, data-analysis tools that are usually only applicable for soluble proteins (without detergents) could be applied. *Ab initio* modelling was performed using *DAMMIF* (Franke & Svergun, 2009 ▸). The only input was a pair distance distribution function *p*(*r*), which was calculated with *DATGNOM* (Petoukhov *et al.*, 2007 ▸) to obtain the correct input data format for *DAMMIF*. No symmetry was assumed and *DAMMIF* was run ten times. Alignment, clustering, selection, averaging and filtering of the ten runs were performed using the automatic algorithm provided in the *ATSAS* online framework (Franke *et al.*, 2017 ▸).

### 2.9. Molecular-weight determination for assessment of the oligomeric state   

The oligomeric state was assessed by comparing the molecular weight (MW) found from SANS with that of the construct (GluA2cryst; 368 kDa). MW was determined from *I*(0) and the concentration (*c*), as well as the average excess scattering length density (Δρ) calculated from the sequence and the average protein density (ρ_p_ = 1.37 g cm^−3^), by MW = [*I*(0)/*c*]·(*N*_A_ρ_p_^2^/Δρ^2^), where *N*_A_ is Avogadro’s number. MW was determined to be close to the expected value (368 kDa) for GluA2 in the AMPA-bound state at pH 7.5 (347 kDa) and GluA2 in the GYKI-bound state (347 kDa). The determined values of MW were, however, unrealistically low for GluA2 in the resting state (220 kDa) and GluA2 in the AMPA-bound state at pH 5.5 (240 kDa). The discrepancies from the expected value of 368 kDa may reflect the uncertainty in the concentration measurements of these proteins. As an alternative approach that is independent of protein concentration measurements, the molecular weight was determined from the scattering invariant *Q* (Porod, 1982 ▸) using the method described by Fischer *et al.* (2010 ▸) (Table 1 ▸, Supplementary Tables S1 and S2) and the method described by Petoukhov *et al.* (2012 ▸) (Supplementary Table S2). Constant backgrounds were determined using Porod plots (Supplementary Fig. S2) when calculating the value of *Q*. As the Fischer method takes the size of the protein into account, it is more precise for the large tetrameric GluA2 protein (368 kDa) than the Petoukhov method (Fischer *et al.*, 2010 ▸). Therefore, MW values obtained using the Fischer method are used throughout the main text and are reported in Table 1 ▸. All values are, however, given in Supplementary Tables S1 and S2.

## 3. Results   

The AMPA receptor GluA2 from rat with deletion of the disordered intracellular C-terminal domain (GluA2cryst; Sobolevsky *et al.*, 2009 ▸) was used to investigate receptor conformations in solution in the resting state (apo), in the presence of the agonist AMPA and in the presence of the negative allosteric modulator GYKI-53655 by the use of SANS with fully matched-out detergent.

### 3.1. GluA2 in the resting state   

SANS data for GluA2 in conditions corresponding to the resting state were obtained in the *q* range from 0.006 to 0.2 Å^−1^. This data set was also reported in a recent publication on contrast-optimized detergents (Midtgaard *et al.*, 2018 ▸) and we showed that the solution structure of GluA2 resembles the X-ray crystal structure. Here, we analyze the data in detail. A flat low-*q* region was observed, as well as no indications of scattering from the detergent molecules around the transmembrane part of the receptor or from free detergent micelles in the data (Midtgaard *et al.*, 2018 ▸). The Fischer analysis yielded a molecular weight of 396 ± 52 kDa, which should be compared with the expected molecular weight of the construct (GluA2cryst; 368 kDa; Table 1 ▸). This indicated that the protein was in the expected tetrameric state, in line with other studies (Dürr *et al.*, 2014 ▸; Yelshanskaya *et al.*, 2016 ▸). A Kratky plot of the data shows that the protein is partially or fully folded (Supplementary Fig. S3).

**Figure 2:**
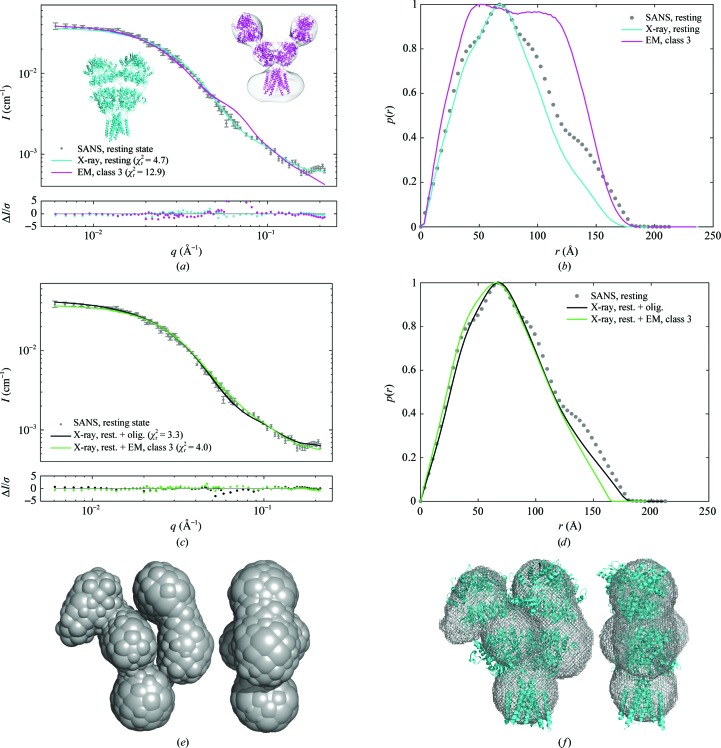
SANS data for GluA2 in the resting state (apo). (*a*) Experimental data (grey points), the resulting fits and residual plots, fitted with the X-ray crystal structure of GluA2 in the resting state (PDB entry 4u2p; cyan; Dürr *et al.*, 2014 ▸) and the EM structure of GluA2 with quisqualate (class 3; EMD-2688; magenta; Meyerson *et al.*, 2014 ▸). A cartoon representation of the crystal structure of GluA2 is shown in cyan and an atomic model fitted into the class 3 EM structure is shown in magenta. (*b*) Pair distance distribution function [*p*(*r*)] for the experimental GluA2 data in solution (grey points) and theoretical *p*(*r*) functions for the GluA2 crystal structure in the resting state (cyan) and for the GluA2 class 3 EM structure (magenta). (*c*) Experimental SANS data and the fit of a linear combination of the crystal structure of GluA2 in the resting state and fractal oligomers (black), and a linear combination of the crystal structure and the class 3 EM structure (green). (*d*) Pair distance distribution functions for the fits. (*e*) *Ab initio* model generated with *DAMMIF*. The size of the beads was weighted by occupancy. (*f*) The GluA2 crystal structure in the resting state (PDB entry 4u2p; cyan) was manually overlaid with the *DAMMIF* envelope (grey).

The GluA2 SANS data were then compared with the 3.2 Å resolution X-ray crystal structure of GluA2 in the resting state (PDB entry 4u2p; Dürr *et al.*, 2014 ▸) by fitting of model 1. This crystal structure of GluA2 in the resting state represents a compact structure (Fig. 1 ▸ *a*). The structure fitted well to the experimental data (with a goodness of fit χ_r_^2^ of 4.7; Fig. 2 ▸ *a*), confirming that the crystal structure was essentially maintained in solution. The desensitized structure of GluA2 with quisqualate (class 3; EMD-2688; Meyerson *et al.*, 2014 ▸) was also incorporated in model 1 and fitted to the experimental data as it represents a structure with the extracellular domains in a more open form (Fig. 1 ▸ *b*). This structure clearly did not fit as well (χ_r_^2^ = 12.9) as the crystal structure of GluA2 in the resting state (Fig. 2 ▸ *a*). This was also confirmed using an *F* test, showing that the difference in the goodness of fit between the resting state and the class 3 EM structure was significant, as the *P* value was below the sigificance level (*P* = 0.0001%, significance level 5%).

The *p*(*r*) function of the GluA2 SANS data was compared with theoretical *p*(*r*) functions calculated from the compact crystal structure of GluA2 in the resting state as well as from the more open class 3 EM structure of GluA2. A plot of experimental data and theoretical curves is displayed in Fig. 2 ▸(*b*). The *p*(*r*) function for the solution GluA2 data had no tail, *i.e.* no indication of oligomerization or aggregation. However, the experimental *p*(*r*) function differed from that from the crystal structure for the resting state by lying above the theoretical function for all distances above *r* ≃ 100 Å. It also had a slightly larger maximal distance (*D*_max_) of 179 ± 11 Å compared with a *D*_max_ of 171 Å based on the crystal structure (Table 1 ▸). Also, the radius of gyration (*R*_g_) was larger for the solution structure (61.9 ± 0.4 Å) compared with the crystal structure (56.1 Å). However, it should be noted that the *D*_max_ and *R*_g_ for the crystal structures are presumably underestimated owing to parts of the TMD not being modelled (Supplementary Fig. S4).

Next, a linear combination of the crystal structure and fractal oligomers (model 2) was fitted to the experimental data, resulting in an even better fit to the data, with χ_r_^2^ = 3.3 for the linear combination compared with χ_r_^2^ = 4.7 for the atomic crystal structure alone. The improvement is minor but significant (*F*-test: *P* = 4.7%, significance level 5%). The fractal oligomers amount to 0.9 ± 6.5%. Note that the amount of fractal oligomers is strongly correlated with the *R*_g_ of the oligomers (Supplementary Table S3) and thus poorly determined (large uncertainty). However, this correlation did not affect the refined values of the remaining model parameters or the goodness of fit. As expected, the inclusion of fractal oligomers improved the fit in the low-*q* region for *q* < 0.02 Å^−1^ (Fig. 2 ▸ *c*). A linear combination of the resting state and the class 3 EM structure (model 3; Fig. 2 ▸ *c*; χ_r_^2^ = 4.0) did not fit significantly better than the crystal structure alone (*F* test: *P* = 22%). Model 4, in which both the crystal structure, fractal oligomers and the class 3 EM structure were included, resulted in the best goodness of fit (χ_r_^2^ = 2.5), but was not statistically better than model 2 (*F*-test: *P* = 10.0%). This suggests that besides the compact structure of GluA2, species with larger dimensions than the X-ray structure of GluA2 in the resting state are present in solution (Fig. 2 ▸ *c*). On the other hand, there is no significant evidence for the presence of a more open conformation like the EM class 3 structure.

An *ab initio* structure was generated using *DAMMIF* (Fig. 2 ▸ *e*). The *ab initio* bead model clearly showed the transmembrane domain and indicated a dimeric arrangement of the ligand-binding domains and the N-terminal domains. The *ab initio* model is similar to the X-ray structure of GluA2 in the resting state but clearly more asymmetric (Fig. 2 ▸ *f*), especially at the NTD level.

### 3.2. GluA2 in the presence of AMPA   

**Figure 3:**
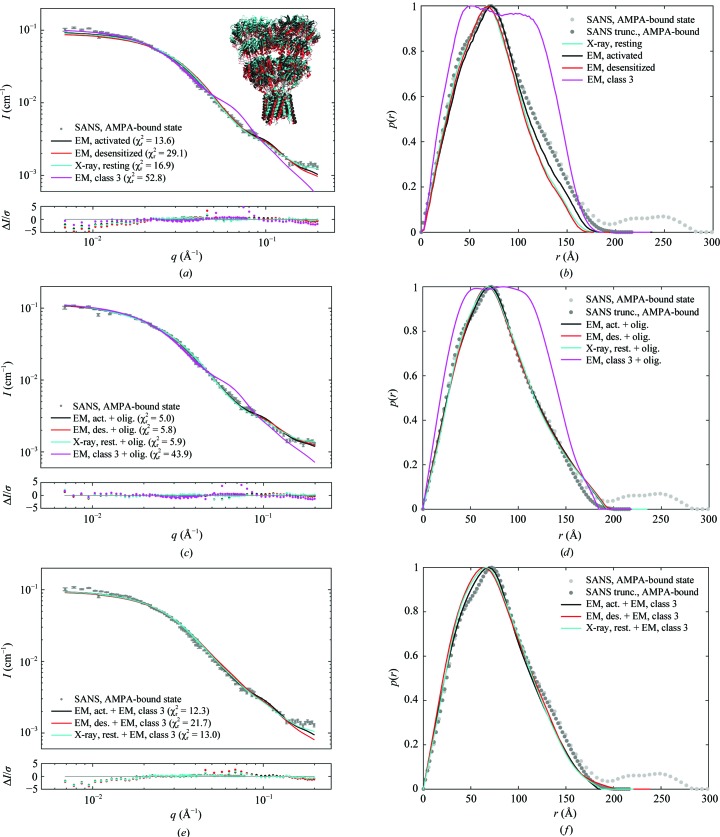
SANS data for GluA2 in the presence of AMPA at pH 7.5. (*a*) Experimental SANS data (grey points) and the resulting structure fits and residual plots for the crystal structure of GluA2 in the resting state (PDB entry 4u2p; cyan; Dürr *et al.*, 2014 ▸), GluA2 in the activated state (PDB entry 5weo; black; Twomey *et al.*, 2017*b* ▸), GluA2 in the desensitized state (PDB entry 5vhz; red; Twomey *et al.*, 2017*a* ▸) and GluA2 in the class 3 EM structure (EMD-2688; magenta; Meyerson *et al.*, 2014 ▸). A cartoon representation of the three structures overlaid is shown in the respective colours. (*b*) *p*(*r*) functions for the SANS data (light grey points) and for a truncated data set (*q* ≥ 0.011 Å^−1^; dark grey points) with the *p*(*r*) for four structures. (*c*, *d*) Resulting fits and *p*(*r*) functions for linear combinations of the atomic structures and fractal oligomers. (*e*, *f*) Resulting fits and *p*(*r*) functions for combinations of the atomic structures and the class 3 EM structure.

SANS data were also collected for GluA2 in the presence of AMPA (Fig. 3 ▸ *a*). As no X-ray crystal or EM structure is available for GluA2 with AMPA, we investigated the data by fitting three different structures: the crystal structure of GluA2 in the resting state, the recently reported structure of GluA2 in the activated state (the cryo-EM structure of GluA2 as a complex bound to glutamate, cyclothiazide and stargazin in digitonin; PDB entry 5weo; Twomey *et al.*, 2017*b* ▸) and the cryo-EM structure of GluA2 in the desensitized state (bound to l-quisqualate and germ cell-specific gene 1-like protein; PDB entry 5vhz; Twomey *et al.*, 2017*a* ▸) (see Fig. 1 ▸ *a*).

The experimental MW based on the SANS data was found to be 379 ± 49 kDa as obtained from Fischer analysis. This is close to the expected MW of 368 kDa for the construct (Table 1 ▸) and consistent with the protein being in a tetrameric state. As for the resting state, the Kratky plot of the data shows that the protein was folded or partially folded (Supplementary Fig. S3). The best fit was obtained with the structure of GluA2 in the activated state (χ_r_^2^ = 13.6; Figs. 3 ▸ *a* and 3 ▸ *b*). The resting state gave a similar fit, although with a slightly worse goodness of fit (χ_r_^2^ = 16.9). However, the goodness of fit was not significantly different between the two structures (*P* = 12.4%). We also fitted structures of GluA2 in the desensitized state (PDB entry 5vhz and class 3 EM), which resulted in even worse fits, with χ_r_^2^ values of 29.1 and 52.8, respectively.

Next, a linear combination of the four structures and fractal oligomers was fitted to the experimental data (model 2; Figs. 3 ▸ *c* and 3 ▸ *d*). The inclusion of fractal oligomers resulted in a marked improvement of the goodness of fit for the activated state (PDB entry 5weo; *P* = 0.0001%), desensitized state (PDB entry 5vhz; *P* = 0.000005%) and resting state (PDB entry 4u2p; *P* = 0.7%), with χ_r_^2^ values of 5.0, 5.8 and 5.9, respectively. However, it was not possible to distinguish among these fits, which is in agreement with the similarity of the structures of GluA2 in the resting, activated and desensitized states, with *D*_max_ in the range 167–175 and an *R*_g_ of 55.8–58.7 (Table 1 ▸). Linear combinations of the resting, activated and desensitized (PDB entry 5vhz) states, respectively, with the open class 3 EM structure (model 3; χ_r_^2^ of 13.0, 12.3 and 21.7, respectively; Figs. 3 ▸ *e* and 3 ▸ *f*) did not fit as well as a linear combination with fractal oligomers (model 2; Figs. 3 ▸ *c* and 3 ▸ *d*). Model 4 was also tested (a linear combination of a compact structure, the loose EM class 3 structure and fractal oligomers). However, using model 4 did not improve the fit significantly compared with model 2 (compact structure and fractal oligomers). These observations support a compact form in solution, combined with a small amount of oligomers of tetrameric GluA2 (approximately 1–2%; see Supplementary Table S3).

**Figure 4:**
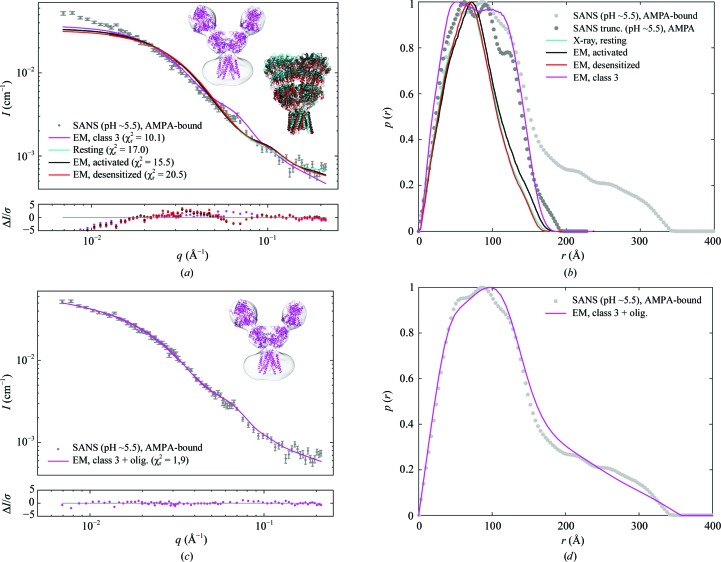
SANS data for GluA2 in the presence of AMPA at pH 5.5. (*a*) Experimental SANS data for GluA2 in the presence of AMPA at pH 5.5 (grey points) and the resulting fits and residual plots of the GluA2 EM structure in the activated state (PDB entry 5weo; black; Twomey *et al.*, 2017*b* ▸), the EM structure in the desensitized state (PDB entry 5vhz; red; Twomey *et al.*, 2017*a* ▸), the X-ray crystal structure in the resting state (PDB entry 4u2p; cyan; Dürr *et al.*, 2014 ▸) and the class 3 EM structure (EMD-2688; magenta; Meyerson *et al.*, 2014 ▸). Cartoon representations of each of the three atomic resolution structures were aligned and are shown in the respective colours. A cartoon representation of the atomic structure fitted to the EM class 3 density map is also shown. (*b*) *p*(*r*) functions for the SANS data (light grey points) and for a truncated data set (*q* ≥ 0.019 Å^−1^; dark grey points) together with the theoretical *p*(*r*) functions for the four structures. (*c*, *d*) Experimental data (nontruncated; grey points), the resulting fit and the *p*(*r*) function for a linear combination of the EM class 3 structure and fractal oligomers (magenta).

When adding ∼10 m*M* AMPA, resulting in an acidic pH of 5.5, we observed a significant structural change (Fig. 4 ▸ and Supplementary Fig. S5). The difference in the SANS data with 10 m*M* AMPA (Fig. 4 ▸) compared with the data with 1 m*M* AMPA (Fig. 3 ▸) is primarily seen in the low-*q* region and in the *q*-range 0.02–0.06 Å^−1^. The calculated molecular weight, estimated by Fischer analysis to be 442 ± 57 kDa, is larger than that of the construct (368 kDa) (Table 1 ▸), but is still in fair agreement with the expected tetrameric state. The Kratky plot showed that the protein was still in a folded or partially folded state (Supplementary Fig. S3). Interestingly, the SANS data at low pH were fitted relatively well by the more open GluA2 class 3 EM structure (Fig. 1 ▸ *b*). The fit of the GluA2 class 3 EM structure resulted in χ_r_^2^ = 10.1, whereas the goodness of fit was worse for the structures of GluA2 in the resting, activated and desensitized states (PDB entry 5vhz; Twomey *et al.*, 2017*a* ▸) (χ_r_^2^ = 17.0, 15.5 and 20.5, respectively; Fig. 4 ▸ *a*). The values of *R*_g_ and *D*_max_ from the *p*(*r*) of the experimental SANS (65.2 ± 0.5 and 189 ± 5 Å) are larger than for GluA2 in the resting state or activated state (Table 1 ▸). On the other hand, these values are in accordance with the theoretical values calculated for the GluA2 class 3 EM structure with a hydration layer (64.1 and 179 Å). When including fractal oligomers in the fit (EM class 3 and fractal oligomers; model 2; 1.0 ± 3.0% oligomers) the goodness of fit was improved significantly (χ_r_^2^ = 1.9), now taking species of larger dimensions into account (Figs. 4 ▸ *c* and 4 ▸ *d*). The data were also fitted with combinations of GluA2 in the resting (χ_r_^2^ = 5.2), activated (χ_r_^2^ = 4.6) and desensitized (χ_r_^2^ = 4.8) states, respectively; all were combined with fractal oligomers (model 2; Supplementary Fig. S6) to check whether a combination of a compact structure and fractal oligomers could explain the data. The obtained χ_r_^2^ values are significantly larger than the χ_r_^2^ of 1.9 obtained for the combination of the loose EM class 3 structure and fractal oligomers. From this, we conclude that the data show the best agreement with the EM class 3 structure, indicating a transition from a compact form to a loose form at low pH and in the presence of AMPA.

### 3.3. GluA2 in the presence of GYKI-53655   

We also investigated the solution structure of GluA2 in the presence of the negative allosteric modulator (noncompetitive antagonist) GYKI-53655. Assessment of the MW by Fischer analysis suggests that the protein is in the tetrameric state, with a calculated MW of 373 ± 48 kDa, which is close to the MW of the construct (368 kDa; Table 1 ▸). The Kratky plot implied a folded or partially folded structure (Supplementary Fig. S3).

**Figure 5:**
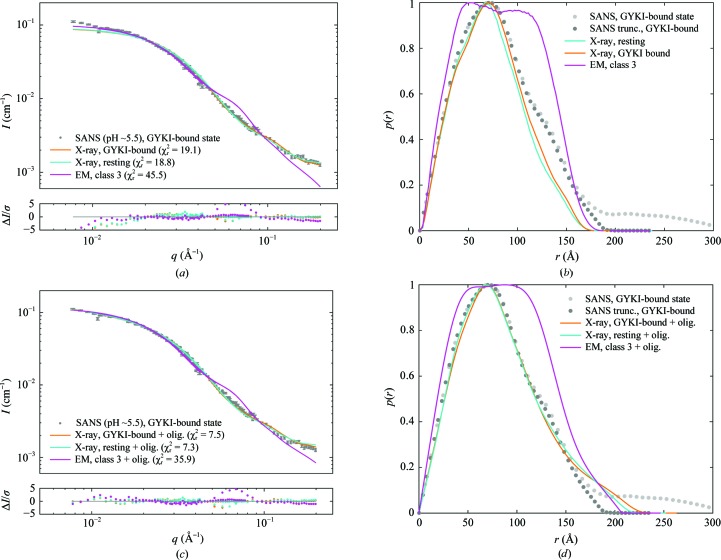
SANS data for GluA2 in the presence of GYKI-53655. (*a*) Experimental SANS data (grey points) and the resulting structure fits and residual plots for the X-ray crystal structures of GluA2 with GYKI-53655 bound (PDB entry 5l1h; orange; Yelshanskaya *et al.*, 2016 ▸), GluA2 in the resting state (PDB entry 4u2p; cyan; Dürr *et al.*, 2014 ▸) and GluA2 in the class 3 EM structure (EMD-2688; magenta; Meyerson *et al.*, 2014 ▸). (*b*) *p*(*r*) functions for the SANS data (light grey) and for a truncated data set (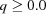 12 Å^−1^; dark grey) and the theoretical *p*(*r*) functions for the two X-ray structures and the class 3 EM structure. (*c*) SANS data (grey points) and fits with a linear combination of the three structures and fractal oligomers. (*d*) *p*(*r*) functions for the SANS data (nontruncated and truncated) and for the linear combinations of the three structures and fractal oligomers.

The SANS data for GluA2 in the presence of GYKI-53655 were fitted by the crystal structure with the same ligand (PDB entry 5l1h; Yelshanskaya *et al.*, 2016 ▸) as well as by the resting state (Fig. 5 ▸ *a*). The goodness of fit was not optimal either for the GluA2 structure with GYKI-53655 (χ_r_^2^ = 19.1) or for GluA2 in the resting state (χ_r_^2^ = 18.8), especially in the low-*q* region. An even worse fit was observed with the class 3 EM structure (χ_r_^2^ = 45.5). Again, including a small amount of fractal oligomers in the fitting procedure (model 2) improved the goodness of fit significantly. For example, when fitting the SANS data using the compact crystal structure of GluA2 with GYKI-53655 the fit was improved from χ_r_^2^ = 19.1 to χ_r_^2^ = 7.5 by the inclusion of 0.2 ± 0.6% fractal oligomers.

## 4. Discussion   

Methods to study the structures of ionotropic glutamate receptors are essential in order to understand how these receptors function as well as to understand their roles in diseases and as targets for medicines. In recent years, the AMPA receptor GluA2 has been thoroughly characterized in the resting, activated and desensitized states using X-ray crystallography and cryo-EM (see, for example, Dürr *et al.*, 2014 ▸; Twomey *et al.*, 2017*a* ▸,*b* ▸; Chen *et al.*, 2017 ▸). In this study, we investigated GluA2 in solution at 10°C using SANS. This was made possible owing to the very recent development of deuterated detergents with separate hydrogen/deuterium balances of the head and tail groups that eliminate all signal from the detergent micelles solubilizing the membrane proteins in deuterated water-based buffer (Midtgaard *et al.*, 2018 ▸). Thus, such detergents allow the direct measurement of the solution structure of the receptor without seeing the surrounding micelles.

Fractal oligomers were included in the fit of atomic structures to the experimental SANS data (models 2 and 4). Ideally, oligomerization in the sample should be avoided, for example by running a SEC-SANS experiment where SANS data are collected *in situ* as the sample leaves the purification column (Jordan *et al.*, 2016 ▸). However, SEC-SANS is still an emerging technique that has only been demonstrated at the D22 instrument at ILL and is generally not a feasible technique for studies with many ligands owing to the large sample consumption of protein, deuterated detergent and ligands. Therefore, the data were instead ‘filtered’ for the scattering contribution from large oligomers by the inclusion of fractal oligomers in the model. The information in the data about the detailed structure of the fractal oligomers is limited, which is reflected in the poorly determined values of γ and *R*_g_ (Supplementary Table S3). These are, however, not the parameters of interest, as the fractal oligomer model merely serves as a mean to minimize misinterpretations owing to the effects of oligomerization. Such models, as well as the associated molecular constraints, constitute a useful tool for future experiments. Combined with the recently developed matched-out detergents (Midtgaard *et al.*, 2018 ▸), this enables the retrieval of information from samples that are not fully monodisperse.

In solution at 10°C, we find that GluA2 primarily adopts a compact tetrameric structure both in the resting state as well as in the presence of 1 m*M* AMPA and 1 m*M* GYKI-53655, resembling the compact X-ray and cryo-EM structures determined at cryogenic temperature (Figs. 2 ▸, 3 ▸ and 5 ▸). Therefore, this study adds support to the observation that GluA2 also preferably adopts a compact conformation under conditions with no restraints on the dynamics of the protein compared with crystal packing in X-ray crystallography and cryogenic temperatures in cryo-EM. This was surprising as the cryo-EM study by Meyerson *et al.* (2014 ▸) showed that GluA2 was more dynamic in the presence of the agonist quisqualate, adopting a range of conformations, of which three were modelled. Furthermore, GluA2 has also previously been shown by cryo-EM to be conformationally heterogeneous in the presence of the partial agonist fluoro­williardiine under desensitizing conditions, suggesting that GluA2 assumes a variety of different conformations (Dürr *et al.*, 2014 ▸). As the X-ray and cryo-EM structures of the resting, activated and desensitized states are very similar (Fig. 1 ▸ *a* and Supplementary Fig. S7), it was statistically not possible to distinguish between these structures when fitted to the SANS data. In all cases, the fits of the compact structures were improved when fitting a linear combination of the atomic structure and small amounts of fractal oligomers, corresponding to a few percent. The *F* test turned out to be a very useful tool for comparing hypothesized models with the SANS data. The *P* values showed that despite minor improvements of the goodness of fit (χ_r_^2^) when fitting different compact structures, or using more complex models, these were not always statistically significant.

It is a characteristic of the X-ray and cryo-EM structures of the resting, activated and desensitized states that they all lack several amino-acid residues in the TMD. Also, the amino-acid sequences are not exactly the same as the sequence used in this study (Supplementary Fig. S4). This might affect the goodness of fit, and especially *R*_g_ and *D*_max_. To address this issue, we introduced the missing amino-acid residues into the X-ray structure of GluA2 in the resting state with *MODELLER* (Fiser *et al.*, 2000 ▸; Webb & Sali, 2014 ▸) using the ‘missing residue’ procedure and assuming a loop structure (Supplementary Fig. S8). This structural model led to an *R*_g_ (60.7 Å) that was more similar to the experimental value but at the same time to a larger *D*_max_ (201.0 Å) than the experimental data, while the deposited structures underestimated *R*_g_ and *D*_max_ owing to missing residues in the structures (Table 1 ▸). The model with inserted loops thus partially explains this discrepancy between the theoretical and experimental scattering, but we do not consider the model to be accurate. Therefore, it was decided to use the deposited structures in the comparisons with the experimental SANS data. It should be noted that including/excluding the missing residues does not change the conclusion that GluA2 forms a compact structure in solution.

An *ab initio* model was generated based on SANS data for GluA2 in the resting state, clearly showing the individual domains: the TMD as well as the extracellular LBD and NTD layers. This *ab initio* model resembles the atomic structure of GluA2, but seems to be more asymmetric than the X-ray and cryo-EM structures. The discrepancy between the *ab initio* model and the crystal structure may, however, be caused by the scattering contribution from the fractal oligomers, since the sample was assumed to be solely in the tetrameric form in the *ab initio* modelling.

It has previously been reported using negative-stain electron microscopy that GluA2 in the resting state adopted 60% compact structure, whereas the addition of 3 m*M* glutamate led to only 3% compact structure (Nakagawa *et al.*, 2005 ▸). This distribution differs from what we observe for GluA2 in solution, where primarily compact structures of GluA2 are seen. We therefore speculated whether the dramatic shift towards more open GluA2 conformations in the negative-stain electron microscopy studies could to some extent be owing to a pH effect, as the use of uranyl acetate typically results in a pH below 5. Interestingly, when measuring GluA2 in the presence of 10 m*M* AMPA, resulting in a pH of 5.5, we observed an increase in the calculated average MW to 442 kDa (Table 1 ▸), which indicated the presence of oligomers in the sample. However, the differences in scattering signals (Figs. 3 ▸ and 4 ▸) on the addition of 10 or 1 m*M* AMPA could not be explained by oligomerization alone. Whereas GluA2 in the presence of 1 m*M* AMPA adopts a compact structure, the SANS data for GluA2 in the presence of 10 m*M* AMPA could be fitted significantly better by a structure with a more open conformation of the extracellular part of GluA2, resembling the class 3 EM structure (Fig. 1 ▸ *b*). Therefore, it is important to consider the impact of ligand concentration and/or pH on the GluA2 structure. As AMPA is present in a large excess compared with GluA2 in this study (∼1000-fold with 1 m*M* AMPA and ∼10 000-fold with 10 m*M*; *K*_d_ of 16.8 n*M*; Couelle *et al.*, 2000 ▸), we suggest that the structural change in GluA2 observed in solution in the presence of 10 m*M* AMPA is primarily owing to a pH effect. Protein stability is well known to be affected by pH, and the structural change could very well be partial unfolding or aggregation. However, given the structural resemblance to the class 3 EM structure, it could be speculated that some ionization-dependent interaction in the extracellular domain was destabilized at this low pH. Interestingly, two histidine residues (His229 in the NTD of chains *B* and *D*; numbering with signal peptide) are located in close proximity on the relatively small interaction surface between the NTDs [417 Å^2^ for GluA2 in the resting state; PDB entry 4u2p; *Protein Interfaces, Surfaces and Assemblies* service (*PISA*) at the European Bioinformatics Institute; http://www.ebi.ac.uk/pdbe/prot_int/pistart.html; Krissinel & Henrick, 2007 ▸]. The p*K_a_* of histidine is most often in the interval 6–7 (Edgcomb & Murphy, 2002 ▸). Therefore, a pH decrease from 7.5 to 5.5 would effectively change the ionization of histidine from neutral to positive, causing repulsion as well as unfavourable interactions with hydrophobic amino-acid residues. Whether this apparent wide-open conformation of GluA2 observed at acidic pH is physiologically relevant is unclear and will require additional studies.

## 5. Conclusion   

In this study we, to the best of our knowledge, report the first data on full-length GluA2 (GluA2cryst with deletion of the CTD) in solution as a detergent-solubilized protein. This was made possible by the recently developed fully matched-out detergent that we have described (Midtgaard *et al.*, 2018 ▸). We show that GluA2 primarily adopts a compact structure in solution at neutral pH both in the resting state as well as in the presence of AMPA or GYKI-53655. Therefore, the solution structures of GluA2 are in accordance with most structures determined by X-ray crystallography and cryo-electron microscopy, but not with the more open class 3 EM structure. This study therefore adds support to the observation that GluA2 also preferably adopts a compact conformation under conditions with no restraints on the dynamics of the protein. Moreover, we observed an altered and more open state at acidic pH in the presence of AMPA that resembes the class 3 EM structure. This observation should stimulate future structural studies. In conclusion, this study can serve as an example for future SANS studies on membrane proteins owing to its methodological focus.

## 6. SASBDB accession codes   

The SANS data and the best fits have been uploaded to the Small Angle Scattering Biological Data Bank (SASBDB; https://www.sasbdb.org; Valentini *et al.*, 2015 ▸) with the following accession codes: SASDDY5 (GluA2 in the resting state), SASDDZ5 (GluA2 in the AMPA-bound state, neutral pH), SASDD26 (GluA2 in the AMPA-bound state, acidic pH) and SASDD36 (GluA2 in the GYKI-53655-bound state).

## 7. Related literature   

The following references are cited in the Supporting Information for this article: Hansen (2000 ▸, 2014 ▸), Konarev *et al.* (2003 ▸) and Tuukkanen *et al.* (2016 ▸).

## Supplementary Material

Supplementary Tables and Figures.. DOI: 10.1107/S2052252518012186/tj5015sup1.pdf

## Figures and Tables

**Table:** (*a*) SANS data.

	*D*_max,SANS_ (Å)	*R*_g,SANS_ (Å)	MW_SANS_ (kDa)	MW_CON_ (kDa)
SANS, resting	179 ± 11	61.9 ± 0.4	396	368
SANS, AMPA-bound (pH 7.5)	184 ± 11	61.0 ± 0.6	379	368
SANS, AMPA-bound (pH 5.5)	189 ± 5	65.2 ± 0.5	442	368
SANS, GYKI-bound	186 ± 5	62.1 ± 0.3	373	368

**Table:** (*b*) Structures.

	*D*_max,THE_ (Å)	*R*_g,THE_ (Å)	MW_STR_ (kDa)
X-ray, resting (PDB entry 4u2p)	171.0	56.1	369
EM, active (PDB entry 5weo)	175.0	58.7	366
EM, desensitized (PDB entry 5vhz)	167.0	55.8	366
EM, class 3 (EMDB-2688)	179.0	64.1	372
X-ray, GYKI-bound (PDB entry 5l1h)	169.5	57.3	359
