# Supplementary material for: Small-angle neutron scattering studies on the AMPA receptor GluA2 in the resting, AMPA-bound and GYKI-53655-bound states
Source: IUCrJ. 2018 Oct 11;5(Pt 6):780–93. doi: 10.1107/S2052252518012186 (PMC6211538; doi:10.1107/S2052252518012186)
Supplement: Supplementary file 1 [file m-05-00780-sup1.pdf]

IUCrJ

Volume 5 (2018)

Supporting information for article:

Small-angle neutron scattering studies on the AMPA receptor GluA2 in the resting, AMPA and GYKI-53655 bound states

Andreas Haahr Larsen, Jerzy Dorosz, Thor Seneca Thorsen, Nicolai Tidemand Johansen, Tamim Darwish, Søren Roi Midtgaard, Lise Arleth and Jette Sandholm Kastrup

Table S1 Information about the samples, the SANS measurements and the software used for the data analysis.

	**GluA2, Apo, in dDDM.**	**GluA2, AMPA-bound state, in dDDM, neutral pH.**	**GluA2, AMPA-bound state, in dDDM, acidic pH.**	**GluA2, GYKI-53655 bound state, in dDDM.**
**Sample details**				
Uniprot ID	P19491 (GRIA2_RAT)			
Organism	*Rattus Norvegicus*			
Ligands	None	1 mM AMPA	10 mM AMPA	1 mM GYKI-53655
Buffer	20 mM Tris/DCl, 100 mM NaCl, 0.5 mM dDDM, pH 7.5	20 mM Tris/DCl, 100 mM NaCl, 0.5 mM dDDM, pH 7.5	20 mM Tris/DCl, 100 mM NaCl, 0.5 mM dDDM, pH 5.5	20 mM Tris/DCl, 100 mM NaCl, 0.5 mM dDDM, pH 7.5
Extinction coefficient*[1] [M^{-1} cm^{-1}]	519100			
Density*[2] [g/ml]	1.37			
Molecular weight*[1] [kDa]	367.7			
Mean scattering length density*[2] of protein in D_2O [10^{-6} Å^{-2}]	3.0			
Mean scattering length density*[2] of DDM tail groups in D_2O [10^{-6} Å^{-2}]	6.4			
Mean scattering length density*[2] of DDM head groups in D_2O [10^{-6} Å^{-2}]	6.4			
Mean scattering length density*[2] of solvent (D_2O) [10^{-6} Å^{-2}]	6.4			
Protein concentration*[3]	0.20 mg/ml 0.54 μM	0.31 mg/ml 0.84 μM	0.17 mg/ml 0.46 μM	0.31 mg/ml 0.84 μM
**SANS data collection details**				
Instrument	KWS1@FRM2 (https://www.mlz-garching.de/kws-1)			
Date for data collection	19/09 2017	8/12 2016	19/09 2017	8/12 2016

Wavelength (λ_{mean}, $\Delta\lambda/\lambda$)	5.0 Å, 10 % (FWHM)			
Beam dimensions	Rectangular beam, 6x10 mm^2 (at sample), 30x30 mm^2 (first pinhole)			
Resolution effects	Width of the resolution function $\Delta q(q)$ was calculated by the beamline software and given in the 4th column in data, which was used in WillItFit.			
Settings (Sample-detector/Collimation)	1.5m/4.0m, 4.0m/4.0m, 8.0m/8.0m			
Measured q-range	0.006-0.3 Å^{-1}			
Absolute calibration	By plexiglass			
Exposure time (total for all 3 settings)	~ 2.5 hours	~ 4.5 hours	~ 4.5 hours	~ 4.0 hours
Temperature	10 °C			
**Software employed**				
Indirect Fourier transformations to obtain $p(r)$	BayesApp[R1,R2] (www.bayesapp.org)			
Calculation of theoretical $p(r)$<br>Addition of water layer to protein	CaPP (https://github.com/Niels-Bohr-Institute-XNS-StructBiophys/CaPP)			
Fitting of data with combined analytical and atomistic models	WillItFit[R3] (https://sourceforge.net/projects/willitfit)			
Fischer/Petoukhov M_W determination	Own implementation in MATLAB (Table S3)			
Missing sequence modelling	MODELLER[R4] (https://salilab.org?modeller)			
Graphic model visualization	PyMOL			
Guinier analysis	Own implementation in MATLAB (Fig. S6)			
Ab initio dummy bead modelling	DAMMIF[R5] (https://www.embl-hamburg.de/biosaxs/dammif.html)<br>Note: DATGNOM[R6,R7] was used to generate the $p(r)$ function needed as input to DAMMIF			
**Structural parameters**				
Guinier analysis				
$I(0)$ [cm^{-1}]	0.043 ± 0.002	0.108 ± 0.004	0.052 ± 0.07*4	0.109 ± 0.004
Mw from $I(0)$ [kDa]	220	347	240	347
(ratio to expected)	(0.60)	(0.94)	(0.66)	(0.94)
Rg [Å]	60.8 ± 4.3	62.2 ± 3.0	79.1 ± 11.5*4	62.8 ± 3.3
Minimum q used [Å^{-1}]	0.0069	0.0078	0.0104	0.0103
Maximum $q \cdot R_g$	1.26	1.24	1.29	1.30
p(r) analysis				

$I(0)$ [cm^{-1}]	0.0444 ± 0.0002	0.106 ± 0.001	0.0418 ± 0.0004	0.109 ± 0.001
Rg [Å]	61.9 ± 0.4	61.0 ± 0.6	65.2 ± 0.5	62.1 ± 0.3
D_{max} [Å]	179 ± 11	184 ± 11	189 ± 5	186 ± 5
Used q-range	[0.011,0.20]	[0.011,0.20]	[0.019,0.21]	[0.012,0.20]
Fitted constant background [cm^{-1}]	0.00052	0.00089	0.00048	0.00090
Reduced χ^2	2.15	6.84	1.39	6.60
Number of good parameters	5.2	4.2	4.1	5.5
Number of Shannon channels	11.8	11.0	11.7	11.0
Number of error calculations	260	759	95	264
Regularization parameter $\log(\alpha)$	14.3	14.7	14.0	14.3
Fischer M_W determination[*5]				
Molecular weight [kDa]	396 ± 52	379 ± 49	442 ± 57	373 ± 48
(ratio to expected)	(1.08)	(1.03)	(1.20)	(1.01)
**Model fitting parameters**				
Combined analytical and atomistic model				
q-range for fitting [Å^{-1}]	[0.006,0.3]	[0.006,0.3]	[0.006,0.3]	[0.006,0.3]
Reduced χ^2 (best fit)	3.3	5.0	1.9	7.5
Ab initio dummy bead modelling				
Number of calculations	10	--*[6]	--*[6]	--*[6]
Symmetry	P1, none			
NSD	1.5 ± 0.1			
Resolution (from SASRES [R8]) [Å]	56 ± 4			
Filtered volume [nm^3]	380			
Mw from filtered volume	238 kDa			
(ratio to expected)	(0.92)			
**SASBDB IDs for data and models**				
SASBDB ID	SASDDY5	SASDDZ5	SASDD26	SASDD36
**Footnotes and references**				

*[1] Calculated with Expasy Protparam (https://web.expasy.org/protparam/).

*[2] Calculated with Biomolecular Scattering Length Density Calculator (http://psldc.isis.rl.ac.uk/Psldc).

*[3] Protein concentration determined by UV280 absorption for GluA2 AMPA-bound at pH 7.5 and GluA2 GYKI-bound. Determined with BCA assay for GluA in the resting state and GluA2 AMPA-bound at pH 5.5.

*[4] It was not possible to obtain fully linear region at $qR_G < 1.3$ (Fig. S6C), so the the values may be incorrect.

*[5] M_W determined with the Fischer method (Fischer *et al.*, 2011) with parameters given in Table S3.

*[6] The dummy atom model for GluA2 apo is approximate, since aggregation was not taken into account. For the same reason, a dummy atom model was only generated for the GluA2 apo sample, where the aggregation scattering contribution was very minor.

[R1] Hansen, S. (2000). *J. Appl. Cryst*. **33**, 1415-1421.

[R2] Hansen, S. (2014). *J. Appl. Cryst*. **47**, 1469-1471.

[R3] Pedersen, M. C., Arleth, L. & Mortensen, K. (2013). *J. Appl. Cryst*. **46**, 1894-1898.

[R4] Fiser, A., Do, R.K. & Sali, A. (2000). *Protein Sci*. **9**, 1753-1773.

[R5] Franke, D. & Svergun, D. I. (2009). *J. Appl. Cryst*. **42**, 342-346.

[R6] Konarev, P. V., Volkov, V. V., Sokolova, A. V., Koch, M. H. J. & Svergun, D. I. (2003). *J. Appl. Cryst*. **36**, 1277-1282.

[R7] Petoukhov, M. V., Konarev, P. V., Kikhney, A. G. & Svergun, D. I. (2007). *J. Appl. Cryst*. **40**, 223-228.

[R8] Tuukkanen, A. T., Kleywegt, G. J. & Svergun, D. I. (2016). *IUCrJ* **3**, 440-447.

Table S2 Fischer and Petoukhov M_W determination (Petoukhov *et al.*, 2012; Fisher *et al.*, 2010), where M_W is determined via. the scattering "invariant" Q (Porod, 1982)

The upper integration limit q_m used to determine Q was $8/R_g$ (Petoukhov *et al.*, 2012). V_{app} is the apparent volume, and is the same for the two methods. In the Fischer method, linear coefficients A and B given in the table are used to convert V_{app} to the Porod volume V_p, and the weight-to-volume conversion constant of 0.83 kDa/nm^3 to obtain M_W^F. In the Petoukhov method, M_W^P is determined directly from the V_{app} using the conversion constant 0.625 kDa/nm^3. The constant subtracted backgrounds K were used to assure a constant plateau in the Porod plots (Fig. S2) and the data sets were extrapolated to $q = 0$ by simple linear extrapolation. An implementation in MATLAB of the methods was used. The value for M_W obtained with the Fisher method is given in Table S1 and used in the paper, since this method takes the size of the particle into account, which adds an important correction for large proteins such as GluA2. Values of R_g and $I(0)$ from the $p(r)$ analysis were used (Table S1).

**Fischer/Petoukhov M_W determination**	**Resting**	**AMPA pH 7.5**[*1]	**AMPA pH 5.5**	**GYKI-53655**
V_{app} [nm^3]	871.7	833.4	970.1	820.4
M_W^F [*1] [kDa]<br>(ratio to expected)<br>$\Delta M_W/\sigma$[*2]	396 ± 52<br>(1.08)<br>0.56	379 ± 49<br>(1.03)<br>0.22	442 ± 57<br>(1.20)<br>1.3	373 ± 48<br>(1.01)<br>0.1
M_W^P * [kDa]<br>(ratio to expected)<br>$\Delta M_W/\sigma$	545 ± 109<br>(1.48)<br>1.5	521 ± 104<br>(1.42)<br>1.5	606 ± 121<br>(2.65)<br>2.8	513 ± 103<br>(1.39)<br>1.4
K [10^{-3}cm^{-1}]	0.65	1.30	0.61	1.30
$q_m = 8/R_g$ [Å^{-1}]	0.133	0.131	0.127	0.131
A [Å^3]<br>B	-10500<br>0.56	-10500<br>0.56	-10500<br>0.56	-10500<br>0.56

[*1] Assuming a 13% uncertainty on M_W^F (Fischer *et al.*, 2010, p. 106), and a 20% uncertainty on M_W^P (Petoukhov *et al.*, 2012, p. 344).

[*2] $\Delta M_W/\sigma$ is the normalized residual molecular weight, i.e. the difference between the experimentally determined value and the expected molecular weight in units of the experimental error. If $M_W/\sigma < 2$ then the null-hypothesis (tetrameric state) cannot be rejected, given a significance level of 5%.

Table S3 R_g of fractal oligomers and the amount of oligomers in the fitted models.

**Data**	**Apo**	**AMPA pH 7.5**			**AMPA pH 5.5**	**GYKI-53655**
Model	X-ray, rest. + frac. olig.	X-ray, rest + frac. olig.	EM, act. + frac. olig.	EM, des. + frac. olig.	EM, class3 + frac. olig.	EM, GYKI + frac. olig.
R_g for fractal oligomers [Å]	126 ± 250	190 ± 177	145 ± 135	116 ± 62	175 ± 166	240 ± 254
Fraction in oligomeric form, γ [%]	0.9 ± 6.5	0.4 ± 1.0	0.7 ± 2.1	2.7 ± 5.3	1.0 ± 3.0	0.2 ± 0.6

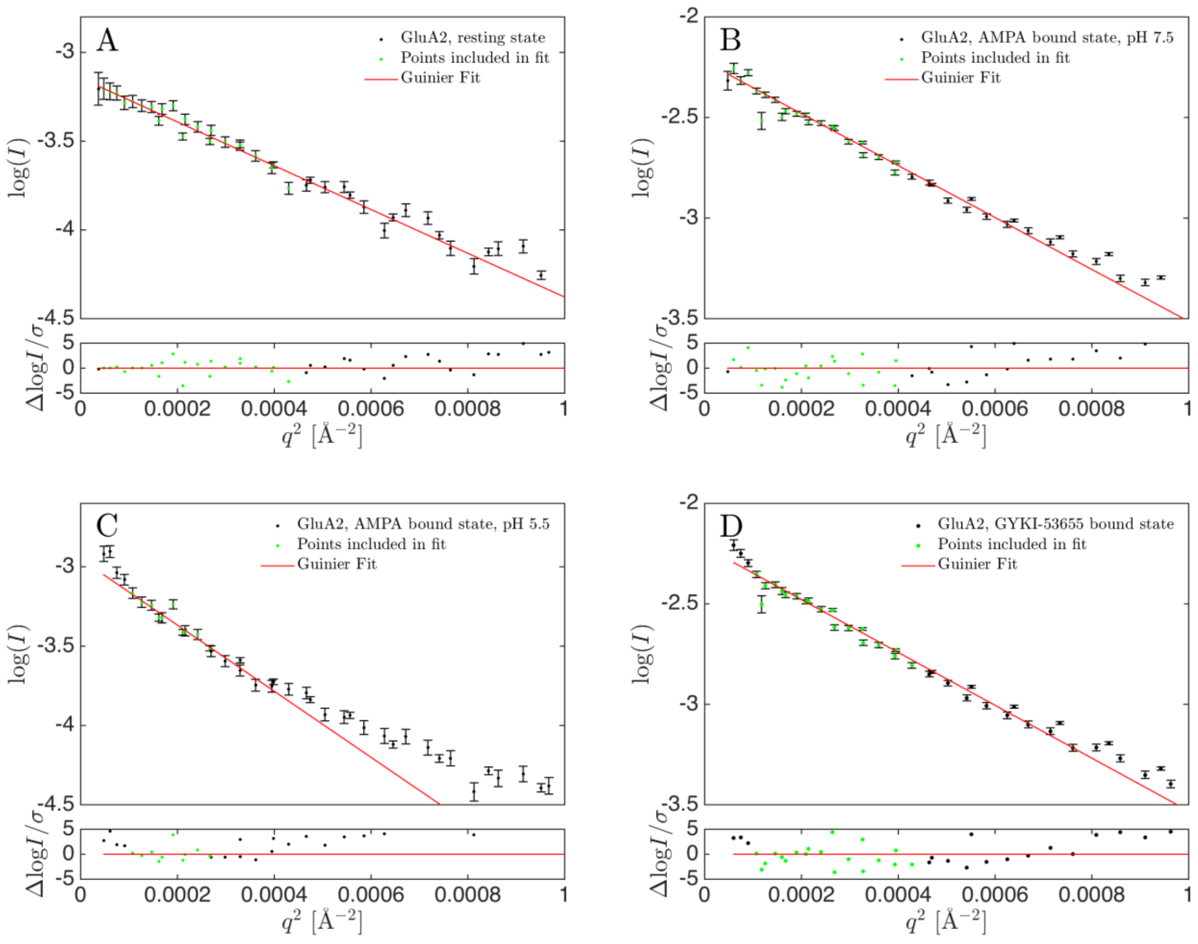

Figure S1 Guinier plots and residual plots for GluA2 in the resting state (A), in the AMPA bound state at pH 7.5 (B), in the AMPA bound state at pH 5.5 (C) and in the GYKI-53655 bound state (D). Residuals show the difference between $\log(I)$ and the fit, weighted with the errors on $\log(I)$. Resulting values for $I(0)$ and R_g are given in Table S1. The AMPA bound state at pH 5.5 (panel C) does not have a fully linear Guinier region at $qR_g < 1.3$, meaning that the values for $I(0)$ and R_g may be wrong. The values of $I(0)$ and R_g from the $p(r)$ funciton was therefore used for M_W determination.

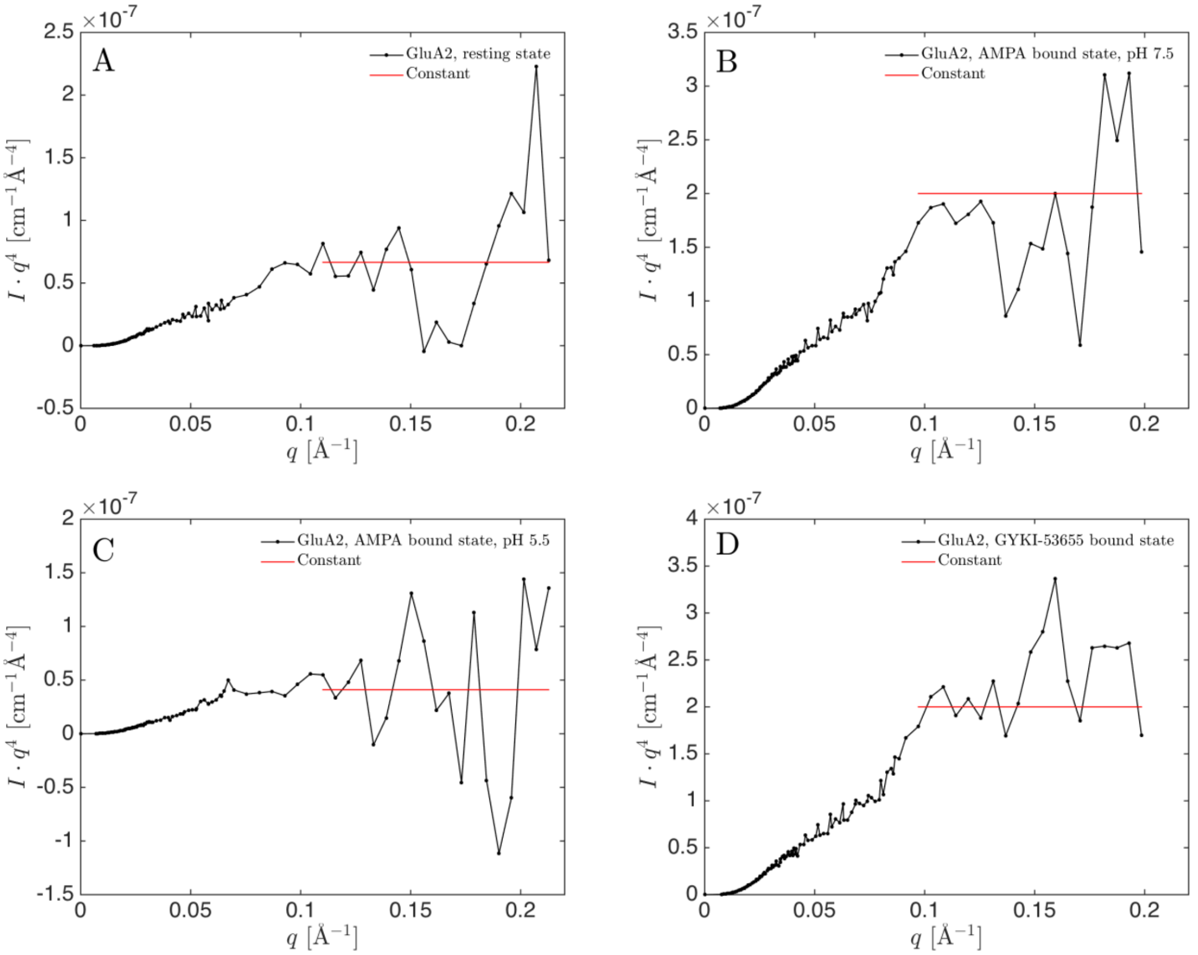

Figure S2 Porod plots (black) for GluA2 in the resting state (A), in the AMPA bound state at pH 7.5 (B), in the AMPA bound state at pH 5.5 (C) and in the GYKI-53655 bound state (D). Additional constant backgrounds were subtracted to give a constant behavior at high-q (red). The constants are listed in Table S2.

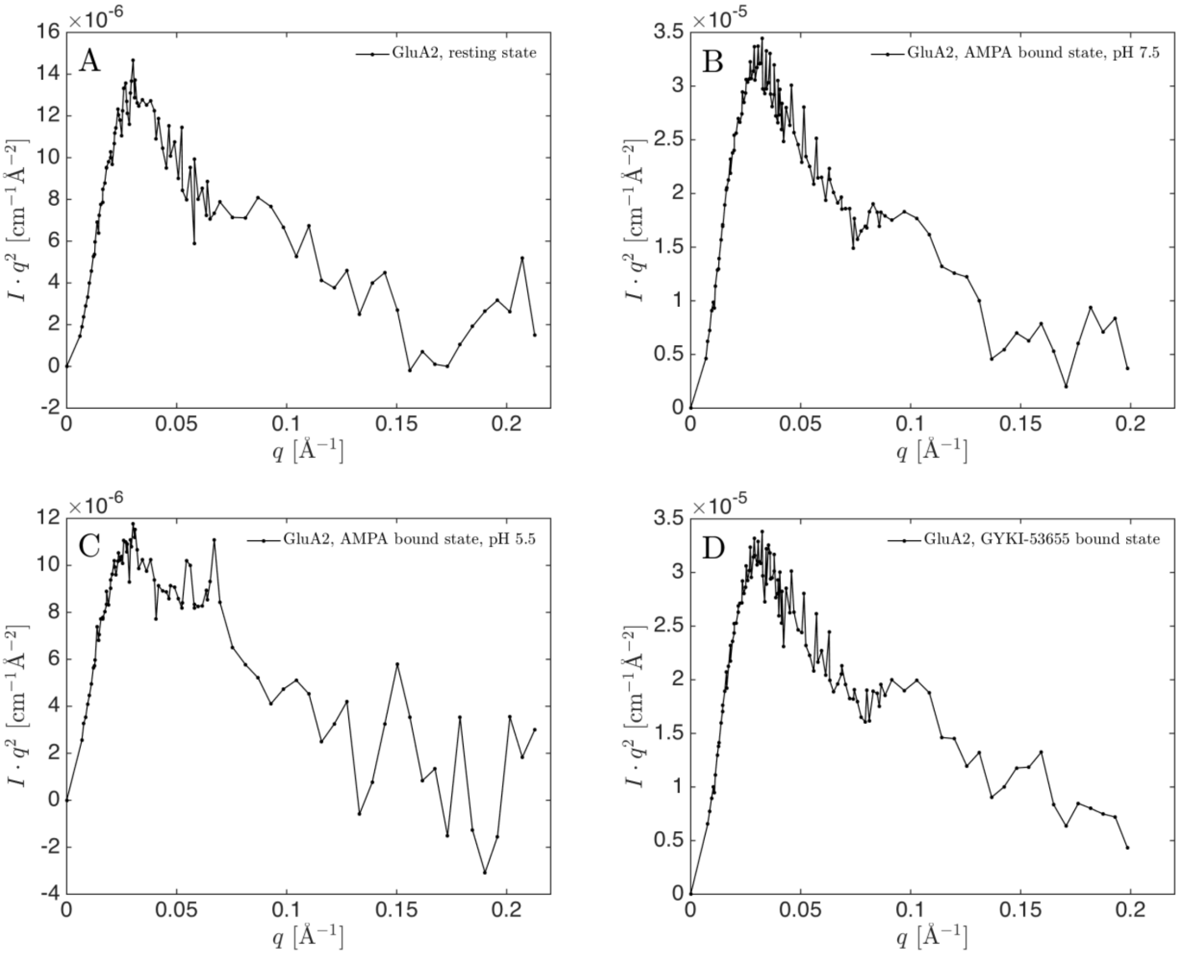

Figure S3 Kratky plots for GluA2 in the resting state (A), in the AMPA bound state at pH 7.5 (B), in the AMPA bound state at pH 5.5 (C) and in the GYKI-53655 bound state (D). Constant backgrounds were subtracted, and listed in Table S2.

3KG2:	NSIQIGGLFPRGADQEYSAFRVGMVQFSTSEFRLTPHIDNLEVANSFAVTNAFCSQFSRG	60
4U2P:	*NS*IQIGGLFPRGADQEYSAFRVGMVQFSTSEFRLTPHIDNLEVANSFAVTNAFCSQFSRG	60
5WEO:	NSIQIGGLFPRGADQEYSAFRVGMVQFSTSEFRLTPHIDNLEVANSFAVTNAFCSQFSRG	60
5VHZ:	NSIQIGGLFPRGADQEYSAFRVGMVQFSTSEFRLTPHIDNLEVANSFAVTNAFCSQFSRG	60
5L1H:	NSIQIGGLFPRGADQEYSAFRVGMVQFSTSEFRLTPHIDNLEVANSFAVTNAFCSQFSRG	60
3KG2:	VYAIFGFYDKKSVNTITSFCGTLHVSFITPSFPTDGTHPFVIQMRPDLKGALLSLIEYYQ	120
4U2P:	VYAIFGFYDKKSVNTITSFCGTLHVSFITPSFPTDGTHPFVIQMRPDLKGALLSLIEYYQ	120
5WEO:	VYAIFGFYDKKSVNTITSFCGTLHVSFITPSFPTDGTHPFVIQMRPDLKGALLSLIEYYQ	120
5VHZ:	VYAIFGFYDKKSVNTITSFCGTLHVSFITPSFPTDGTHPFVIQMRPDLKGALLSLIEYYQ	120
5L1H:	VYAIFGFYDKKSVNTITSFCGTLHVSFITPSFPTDGTHPFVIQMRPDLKGALLSLIEYYQ	120
3KG2:	WDKFAYLYDSDRGLSTLQAVLDSAAEKKWQVTAINVGNINNDKKDETYRSLFQDLELKKE	180
4U2P:	WDKFAYLYDSDRGLSTLQAVLDSAAEKKWQVTAINVGNINNDKKDETYRSLFQDLELKKE	180
5WEO:	WDKFAYLYDSDRGLSTLQAVLDSAAEKKWQVTAINVGNINNDKKDETYRSLFQDLELKKE	180
5VHZ:	WDKFAYLYDSDRGLSTLQAVLDSAAEKKWQVTAINVGNINNDKKDETYRSLFQDLELKKE	180
5L1H:	WDKFAYLYDSDRGLSTLQAVLDSAAEKKWQVTAINVGNINNDKKDETYRSLFQDLELKKE	180
3KG2:	RRVILDCERDKVNDIVDQVITIGKHVKGYHYIIANLGFTDGDLLKIQFGGAEVSGFQIVD	240
4U2P:	RRVILDCERDKVNDIVDQVITIGKHVKGYHYIIANLGFTDGDLLKIQFGGAEVSGFQIVD	240
5WEO:	RRVILDCERDKVNDIVDQVITIGKHVKGYHYIIANLGFTDGDLLKIQFGGAEVSGFQIVD	240
5VHZ:	RRVILDCERDKVNDIVDQVITIGKHVKGYHYIIANLGFTDGDLLKIQFGGAEVSGFQIVD	240
5L1H:	RRVILDCERDKVNDIVDQVITIGKHVKGYHYIIANLGFTDGDLLKIQFGGAEVSGFQIVD	240
3KG2:	YDDSLVSKFIERWSTLEEKEYPGAHTATIKYTSALTYDAVQVMTEAFRNLRKQRIEISRR	300
4U2P:	YDDSLVSKFIERWSTLEEKEYPGAHTATIKYTSALTYDAVQVMTEAFRNLRKQRIEISRR	300
5WEO:	YDDSLVSKFIERWSTLEEKEYPGAHTATIKYTSALTYDAVQVMTEAFRNLRKQRIEISRR	300
5VHZ:	YDDSLVSKFIERWSTLEEKEYPGAHTATIKYTSALTYDAVQVMTEAFRNLRKQRIEISRR	300
5L1H:	YDDSLVSKFIERWSTLEEKEYPGAHTATIKYTSALTYDAVQVMTEAFRNLRKQRIEISRR	300
3KG2:	GNAGDCLANPAVPWGQGVEIERALKQVQVEGLSGNIKFDQNGKRINYTINIMELKTNGPR	360
4U2P:	GNAGDCLANPAVPWGQGVEIERALKQVQVEGLSGNIKFDQNGKRINYTINIMELKTNGPR	360
5WEO:	GNAGDCLANPAVPWGQGVEIERALKQVQVEGLSGNIKFDQNGKRINYTINIMELKTNGPR	360
5VHZ:	GNAGDCLANPAVPWGQGVEIERALKQVQVEGLSGNIKFDQNGKRINYTINIMELKTNGPR	360
5L1H:	GNAGDCLANPAVPWGQGVEIERALKQVQVEGLSGNIKFDQNGKRINYTINIMELKTNGPR	360
3KG2:	KIGYWSEVDKMV--LTEDDTSGLEQKTVVVTTILESPYVMMKANHAALAGNERYEGYCVD	418
4U2P:	KIGYWSEVDKMVVTLTEDDTSGLEQKTVVVTTILESPYVMMKKNHEMLEGNERYEGYCVD	420
5WEO:	KIGYWSEVDKMV--LTEDDTSGLEQKTVVVTTILESPYVMMKKNHEMLEGNERYEGYCVD	418
5VHZ:	KIGYWSEVDKMV--LTEDDTSGLEQKTVVVTTILESPYVMMKKNHEMLEGNERYEGYCVD	418
5L1H:	KIGYWSEVDKMV--LTEDDTSGLEQKTVVVTTILESPYVMMKKNHEMLEGNERYEGYCVD	418

3KG2:	LAAEIAKHCGFKYKLTIVGDGKYGARDADTKIWNGMVGELVYGKADIAIAPLTITLVREE	478
4U2P:	LAAEIAKHCGFKYKLTIVGDGKYGARDADTKIWNGMVGELVYGKADIAIAPLTITLVREE	480
5WEO:	LAAEIAKHCGFKYKLTIVGDGKYGARDADTKIWNGMVGELVYGKADIAIAPLTITLVREE	478
5VHZ:	LAAEIAKHCGFKYKLTIVGDGKYGARDADTKIWNGMVGELVYGKADIAIAPLTITLVREE	478
5L1H:	LAAEIAKHCGFKYKLTIVGDGKYGARDADTKIWNGMVGELVYGKADIAIAPLTITLVREE	478
3KG2:	VIDFSKPFMSLGISIMIKKPQKSKPGVFSFLDPLAYEIWMCIVFAYIGVSVVLFLVSRFS	538
4U2P:	VIDFSKPFMSLGISIMIKKPQKSKPGVFSFLDPLAYEIWMAIVFAYIGVSVVLFLVSR*FS*	540
5WEO:	VIDFSKPFMSLGISIMIKKPQKSKPGVFSFLDPLAYEIWMCIVFAYIGVSVVLFLVSRFS	538
5VHZ:	VIDFSKPFMSLGISIMIKKPQKSKPGVFSFLDPLAYEIWMCIVFAYIGVSVVLFLVS*RFS*	538
5L1H:	VIDFSKPFMSLGISIMIKKPQKSKPGVFSFLDPLAYEIWMCIVFAYIGVSVVLFLVSD--	536
3KG2:	PYEWHTEEFEDGRETQSSESTNEFGIFNSLWFSLGAFMQQGADISPRSLSGRIVGGVWWF	598
4U2P:	*PYEWHTEEFEDGRETQSSESTNEFGIFNSLWFSLGAFFQQGADIS*PRSLSARIVAGVWWF	600
5WEO:	PY*EWHTEEFEDGRETQS*SESTNEFGIFNSLWFSLGAFMQQGCDISPRSLSGRIVGGVWWF	598
5VHZ:	*PYEWHTEEFEDGRETQSSESTNEFG*IFNSLWFSLGAFMQQGCDISPRSLSGRIVGGVWWF	598
5L1H:	-----------------*TDSTNEFG*IFNSLWFSLGAFMQQG*ADISPR*SLSGRIVGGVWWF	579
3KG2:	FTLIIISSYTANLAAFLTVERMVSPIESAEDLSKQTEIAYGTLDSGSTKEFFRRSKIAVF	658
4U2P:	FTLIIISSYTANLAAFLTVERMVSPIESAEDLSKQTEIAYGTLDSGSTKEFFRRSKIAVF	660
5WEO:	FTLIIISSYTANLAAFLTVERMVSPIESAEDLSKQTEIAYGTLDSGSTKEFFRRSKIAVF	658
5VHZ:	FTLIIISSYTANLAAFLTVERMVSPIESAEDLSKQTEIAYGTLDSGSTKEFFRRSKIAVF	658
5L1H:	FTLIIISSYTANLAAFLTVERMVSPIESAEDLSKQTEIAYGTLDSGSTKEFFRRSKIAVF	639
3KG2:	DKMWTYMRSAEPSVFVRTTAEGVARVRKSKGKYAYLLESTMNEYIEQRKPCDTMKVGGNL	718
4U2P:	DKMWTYMRSAEPSVFVRTTAEGVARVRKSKGKYAYLLESTMNEYIEQRKPCDTMKVGGNL	720
5WEO:	DKMWTYMRSAEPSVFVRTTAEGVARVRKSKGKYAYLLESTMNEYIEQRKPCDTMKVGGNL	718
5VHZ:	DKMWTYMRSAEPSVFVRTTAEGVARVRKSKGKYAYLLESTMNEYIEQRKPCDTMKVGGNL	718
5L1H:	DKMWTYMRSAEPSVFVRTTAEGVARVRKSKGKYAYLLESTMNEYIEQRKPCDTMKVGGNL	699
3KG2:	DSKGYGIATPKGSSLGTPVNLAVLKLSEQGLLDKLKNKWWYDKGECGAKDSGSKEKTSAL	778
4U2P:	DSKGYGIATPKGSSLGTPVNLAVLKLSEQGVLDKLKNKWWYDKGECGAKDSGSKEKTSAL	780
5WEO:	DSKGYGIATPKGSSLGTPVNLAVLKLSEQGVLDKLKNKWWYDKGECGAKDSGSKEKTSAL	778
5VHZ:	DSKGYGIATPKGSSLGTPVNLAVLKLSEQGVLDKLKNKWWYDKGECGAKDSGSKEKTSAL	778
5L1H:	DSKGYGIATPKGSSLGTPVNLAVLKLSEQGVLDKLKNKWWYDKGECGAKDSGSKEKTSAL	759
3KG2:	SLSNVAGVFYILVGGLGLAMLVALIEFCYKSRAEAKRMKGLVPRG	823
4U2P:	SLSNVAGVFYILVGGLGLAMLVAL*IEFAYKSRAEAKRMKGLVPR*-	824
5WEO:	SLSNVAGVFYILVGGLGLAMLVALIEFCYKSRA*EAKRMK*------	817
5VHZ:	SLSNVAGVFYILVGGLGLAMLVALIEFCYKSRA*EAKRMK*------	817
5L1H:	SLSNVAGVFYILVGGLGLAMLVALIEFCY*KSRAEAKRMKGLVPR*-	803

Figure S4 Sequence alignment of GluA2 structures used in present study. The alignment was made using Clustal Omega (Goujon, M., McWilliam. H., Li, W., Valentin, F., Squizzato, S., Paern, J. & Lopez, R. A new bioinformatics analysis tools framework at EMBL-EBI (2010) *Nucl. Acids Res*. W695-699). Residues in green are differing from the target sequence (3kg2). The residues marked in italics were not seen in the structures (for chain A, similar for the other chains).

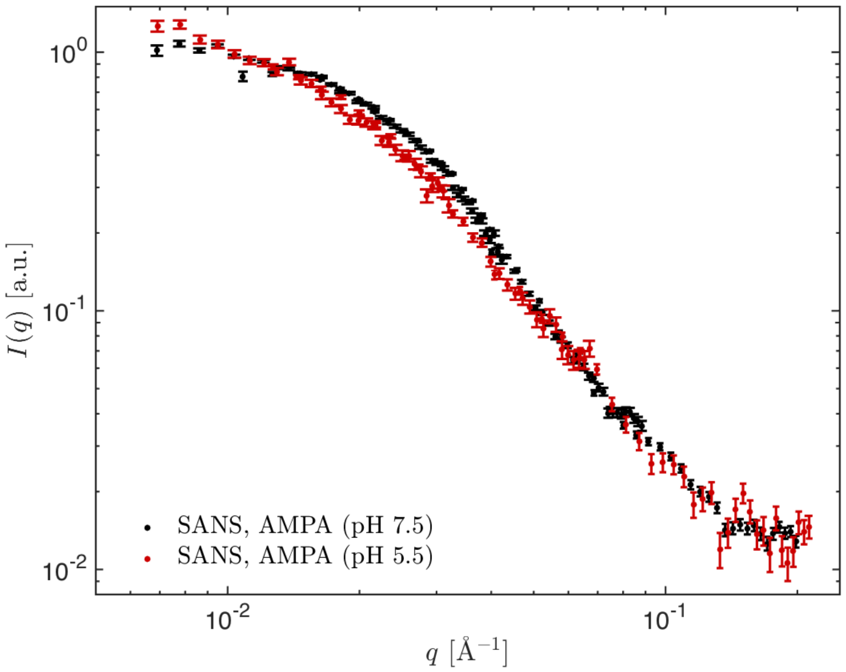

Figure S5 SANS data of GluA2 in the presence of 1 mM AMPA at pH 7.5 (black) and 10 mM AMPA at pH 5.5 (red)

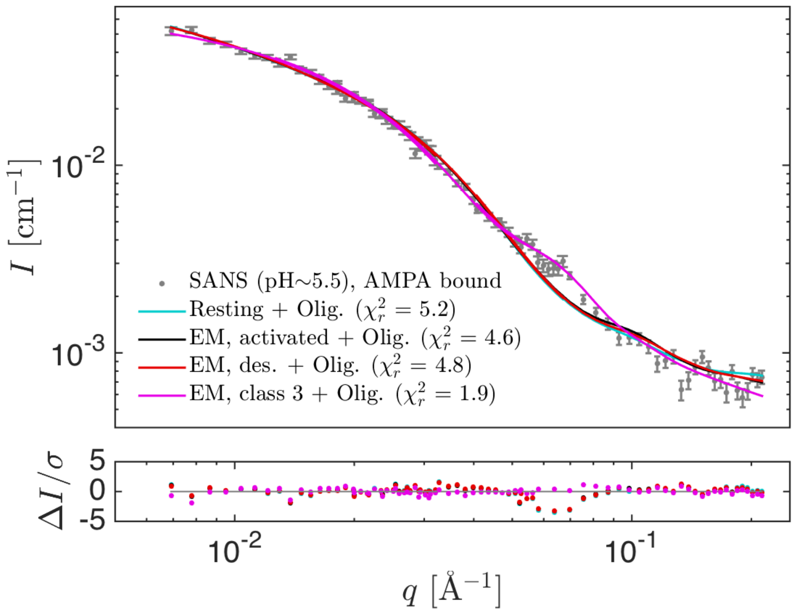

Figure S6 Additional fits to SANS data of GluA2 in the AMPA bound state at pH 5.5 (grey). The data were fitted with models of tetrameric GluA2 in combination with fractal oligomers. Models included GluA2 in the resting state (cyan, pdb-code 4u2p, $\chi_r^2 = 5.2$), GluA2 in the activated state (black; pdb-code 5weo; $\chi_r^2 = 4.6$), GluA2 in the desensitized state (red; pdb-code 5lhv; $\chi_r^2 = 4.8$) and the class 3 EM structure (magenta; EMD-2688; $\chi_r^2 = 1.9$).

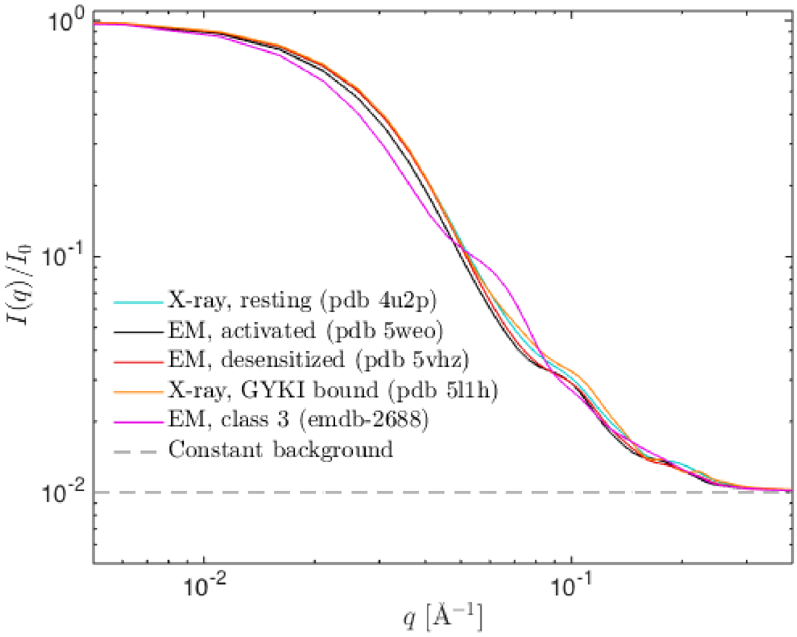

Figure S7 Theoretical SANS scattering for all investigated structures. GluA2 in the resting state (X-ray; cyan; pdb-code 4u2p), in the activated state (EM; black; pdb-code 5weo), in the desensitized state (EM; pdb-code 5vhz), in the GYKI-53655 bound state (X-ray; orange; pdb-code 5l1h) and GluA2 in the class 3 state (EM; magenta; EMDB-2688). Data are normalized and a constant background of $0.01 \cdot I(0)$ is subtracted (grey dashed line). The compact forms are similar, whereas the scattering curve for the more open EM class 3 structure is clearly distinguishable by eye. The compact structures differs only at high q-values, where the the signal to noise ratio is low.

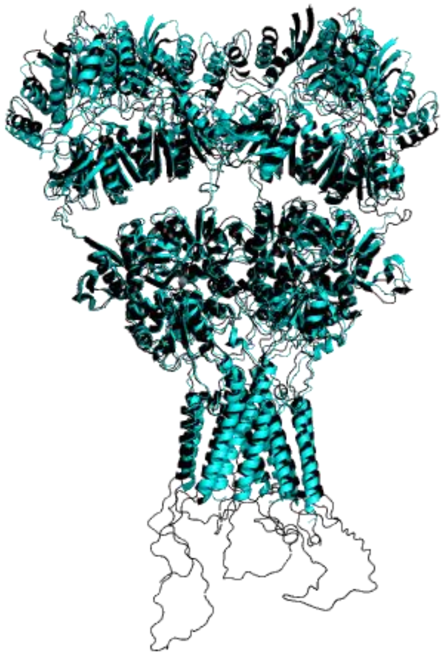

Figure S8 Generated structure of GluA2 in the resting state. Due to missing residues in the X-ray structure of GluA2 in the resting state (cyan; pdb-code 4u2p), a model structure was generated of GluA2 (black) using Modeller (Fiser et al., 2000), with the missing residues inserted as loops.

References

Fischer, H., de Oliveira Neto, M., Napolitano, H. B., Polikarpov, I. & Craievich, A. F. (2010). *J. Appl. Cryst.* **43**, 101-109.

Fiser, A., Do, R.K.G. & Šali, A. (2000). *Protein Sci.* **9**, 1753-1773.

Petoukhov, M. V., Franke, D., Shkumatov, A. V., Tria, G., Kikhney, A. G., Gajda, M., Gorba, C., Mertens, H. D., Konarev, P.V. & Svergun, D. I. (2012). *J. Appl. Cryst.* **45**, 342-350.

Porod, G. (1982). *Small Angle X-ray Scattering* (Glatter, O. & Kratky, O., ed.), New York: Academic Press, Chapter 2, 17-52.
